# A multi-level study of recombinant *Pichia pastoris *in different oxygen conditions

**DOI:** 10.1186/1752-0509-4-141

**Published:** 2010-10-22

**Authors:** Kristin Baumann, Marc Carnicer, Martin Dragosits, Alexandra B Graf, Johannes Stadlmann, Paula Jouhten, Hannu Maaheimo, Brigitte Gasser, Joan Albiol, Diethard Mattanovich, Pau Ferrer

**Affiliations:** 1 Department of Chemical Engineering, Autonomous University of Barcelona, Spain; 2 Institute of Applied Microbiology, Department of Biotechnology, University of Natural Resources and Applied Life Sciences, Vienna, Austria; 3 School of Bioengineering, University of Applied Sciences, FH Campus Vienna, Austria; 4 Department of Chemistry, University of Natural Resources and Applied Life Sciences, Vienna, Austria; 5 VTT Technical Research Centre of Finland, Espoo, Finland

## Abstract

**Background:**

Yeasts are attractive expression platforms for many recombinant proteins, and there is evidence for an important interrelation between the protein secretion machinery and environmental stresses. While adaptive responses to such stresses are extensively studied in *Saccharomyces cerevisiae*, little is known about their impact on the physiology of *Pichia pastoris*. We have recently reported a beneficial effect of hypoxia on recombinant Fab secretion in *P. pastoris *chemostat cultivations. As a consequence, a systems biology approach was used to comprehensively identify cellular adaptations to low oxygen availability and the additional burden of protein production. Gene expression profiling was combined with proteomic analyses and the ^13^C isotope labelling based experimental determination of metabolic fluxes in the central carbon metabolism.

**Results:**

The physiological adaptation of *P. pastoris *to hypoxia showed distinct traits in relation to the model yeast *S. cerevisiae*. There was a positive correlation between the transcriptomic, proteomic and metabolic fluxes adaptation of *P. pastoris *core metabolism to hypoxia, yielding clear evidence of a strong transcriptional regulation component of key pathways such as glycolysis, pentose phosphate pathway and TCA cycle. In addition, the adaptation to reduced oxygen revealed important changes in lipid metabolism, stress responses, as well as protein folding and trafficking.

**Conclusions:**

This systems level study helped to understand the physiological adaptations of cellular mechanisms to low oxygen availability in a recombinant *P. pastoris *strain. Remarkably, the integration of data from three different levels allowed for the identification of differences in the regulation of the core metabolism between *P. pastoris *and *S. cerevisiae*. Detailed comparative analysis of the transcriptomic data also led to new insights into the gene expression profiles of several cellular processes that are not only susceptible to low oxygen concentrations, but might also contribute to enhanced protein secretion.

## Background

Over the last two decades significant progress has been made in heterologous protein production, particularly due to the initiation of the genomics era. The acquisition of profound knowledge and entire genome sequences of a number of expression platforms has lead to considerable success for the production of many pharmaceutical proteins or industrial enzymes [1-3]. Nevertheless, understanding the mechanisms governing efficient production of very complex proteins as functional entities still remains a major challenge.

Over the past recent years, it has been demonstrated that correct protein folding and secretion are highly interrelated with environmental stress factors [4]. While adaptive responses to such stresses have been studied in the model yeast *Saccharomyces cerevisiae *[5,6] little is known about their influence on the physiology of *P. pastoris*. Furthermore, such studies have generally not been performed under heterologous protein production conditions. Only recently, Dragosits and co-workers [7] described the impact of temperature on the proteome of recombinant *Pichia pastoris *in a chemostat-based study. Their data indicated that a decreased folding stress at lower cultivation temperatures (20°C) considerably favoured heterologous Fab antibody production. Some other studies described a similar effect of temperature on the yield of recombinant proteins in other hosts [8,9], but without much information on the underlying physiological mechanisms. Even less is known about the impact of oxygen availability on the physiology of recombinant yeasts. Oxygen limitation strongly affects the core metabolism by causing energy deprivation. Cells have to shift growth to biomass reorganization in order to cope with the strongly reduced availability of ATP, and readjust their metabolic fluxes from cellular respiration to fermentation. The extent of such readjustment is certainly an important issue in facultative anaerobe yeasts with different capacities to ferment glucose (Crabtree effect). *P. pastoris *is 'Crabtree-negative' and more sensitive to the availability of oxygen than the 'Crabtree-positive' *S. cerevisiae*. Since the protein expression machinery is a multistep metabolic process that requires ATP, a shift to fermentative metabolism could also impact on the protein synthesis and/or secretion processes. While affecting growth and protein production, oxygen also influences cellular redox reactions, and these are interlinked with protein folding reactions within the cell. Moreover, protein folding related oxidative stress has been described [10-12].

Paradoxically, we have recently demonstrated that hypoxic conditions in chemostat as well as fed batch cultures significantly increased the specific productivity of recombinant *P. pastoris *[13]. This yeast expression system has become increasingly popular as it represents a valuable and cost-effective tool for protein engineering studies with potential of performing many of the posttranslational modifications typically associated with higher eukaryotes (reviewed in [14]). However, it is not straightforward to predict whether this "hypoxic effect" would be also observed in other expression systems like *S. cerevisiae*, as yeasts behave in a different way with regard to their capacity to secrete, to process and to modify proteins in particular cases. As a consequence, it is important to systematically identify the complex mechanisms ruling efficient protein production, and integrated 'omics' studies are a valuable tool for the study of biological processes. An appropriate cultivation system that allows for strictly controlled environmental conditions while changing one single parameter, like it is the case for chemostat cultivations, provides an excellent basis for such a systems biology approach. Of particular importance is the maintenance of a constant specific growth rate in order to avoid growth rate related effects in the data [15].

In the present study, an integrative multilevel analysis of the physiological response to oxygen availability in *P. pastoris*, with emphasis on its interaction with cellular processes involved in the recombinant expression of an antibody Fab fragment, has been performed in carbon-limited chemostat cultivations with a fixed growth rate at different oxygenation rates. Despite the availability of the strong methanol-inducible alcohol oxidase promoter (AOX) that is extensively used for foreign protein production in this methylotrophic yeast, we opted for the constitutive expression of our model protein (Fab) under the control of the glycolytic glyceraldehyde-3-phosphate dehydrogenase GAP (TDH3) promoter. The use of glucose as sole carbon source facilitates easier handling of a continuous cultivation, reduced heat production and oxygen demand, as well as minimizing cell viability loss and protease release [16]. Furthermore, the use of the GAP (TDH3) promoter allows a direct comparison with other parallel studies on temperature and osmolarity [7,17].

The aim of this work was to exploit the potential of a multi-level study integrating transcriptomic, proteomic and metabolic flux analyses as a powerful tool to comprehensively understand the physiological adaptation of *P. pastoris *to oxygen availability, as well as unravelling potentially distinct features of such adaptation as described for *S. cerevisiae*. In addition, the study pursues leading to new insights into the global mechanisms connecting protein production to environmental conditions, in particular those that lead to increased product formation under hypoxia, as previously reported [13].

## Results and Discussion

In order to study the global adaptive response of recombinant *Pichia pastoris *to oxygen availability, we integrated transcriptome, proteome and metabolic flux data of cells grown in steady state cultures under normoxic and oxygen-limiting, and pseudo-steady state cultures under hypoxic conditions (see Material and Methods). Special emphasis was given to the comparison between fully aerobic (normoxic) and hypoxic conditions, given that this shift contributed to increased recombinant product secretion [13]. The macroscopic growth parameters for both control and Fab-producing *P. pastoris *strains growing under the three different oxygen setpoints have been recently reported elsewhere [13,18]. A summary of these data for the expressing strain is given in Table 1.

**Table 1:** Overview of the macroscopic growth parameters

**O**_**2**_ [%]	YDM **[g L**^**-1**^**]**	biomass yield **[g**_**YDM **_**g**_**glc**_^**-1**^**]**	**specific Fab conc**. **[mg**_**Fab3H6 **_**g**_**YDM**_^**-1**^**]**	ethanol **[g L**^**-1**^**]**	arabitol **[g L**^**-1**^**]**	OUR **[mmol g**_**YDM**_^**-1 **^**h**^**-1**^**]**	CER **[mmol g**_**YDM**_^**-1 **^**h**^**-1**^**]**
21	23.98 ± 1.16	0.47 ± 0.02	0.22 ± 0.01	-	-	2.23 ± 0.03	2.34 ± 0.04
11	22.54 ± 1.83	0.45 ± 0.01	0.38 ± 0.01	0.89 ± 0.16	0.90 ± 0.22	2.26 ± 0.16	2.57 ± 0.16
8	12.58 ± 1.94	0.25 ± 0.01	0.54 ± 0.02	6.85 ± 0.33	2.88 ± 0.31	4.14 ± 0.21	5.62 ± 0.32

Physiological parameters of the 3H6 Fab producing strain grown at normoxic (21%), oxygen-limiting (11%) and hypoxic (8%) conditions in glucose-limited chemostat cultures at D = 0.1 h^-1^. Values represent the mean ± standard error from three biological replicas. YDM = yeast dry mass; glc = glucose; nd = not detected; OUR = oxygen uptake rate; CER = carbon dioxide evolution rate.

### Global transcriptional adaptation of recombinant *Pichia pastoris *to hypoxia

The global transcriptional profile of *P. pastoris *grown in different conditions of oxygen availability was studied with samples from three individual chemostat cultivations each. At a first glance, the microarray statistics (see Table 2) of the approximately 3900 annotated sequences for *P. pastoris *displayed more than 600 genes, independent of the strain, that were differently regulated (adjusted *p*-value ≤ 0.05) when comparing normoxic and hypoxic conditions. A log2 fold change threshold of 0.59 (equivalent to a 1.5 fold change) was not included in this statistics given that also small changes in the gene expression were considered to be crucial for a global view (*e.g*., for Gene Ontology GO term clustering).

**Table 2:** Microarray statistics

3904 annotated sequences, *p *≥ 0.05, no log2 fold change				
**comparison**	**threshold passed**	**up**	**down**	**% regulated**

**C 8/21**	656	357	299	16.80

**C 11/21**	504	261	243	12.91

**C 8/11**	4	2	2	0.10

**E 8/21**	649	342	307	16.62

**E 11/21**	349	172	177	8.94

**E 8/11**	157	62	95	4.02

**E/C 8**	6	4	2	0.15

**E/C 11**	0	0	0	0.00

**E/C 21**	3	2	1	0.08

Microarray statistics including all genes that passed the adjusted *p*-value threshold (*p*-value ≤ 0.05). Comparisons between oxygen setpoints within one strain (e.g. C 8/21) or between strains at a certain oxygen setpoint (E/C 8) are given. For pairwise comparisons, the type of gene regulation (up- or downregulated) always refers to the lower oxygen setpoint or to the expressing strain. C = control strain; E = expressing strain; 8 = hypoxia; 11 = oxygen limitation; 21 = normoxia

**Figure 1:**
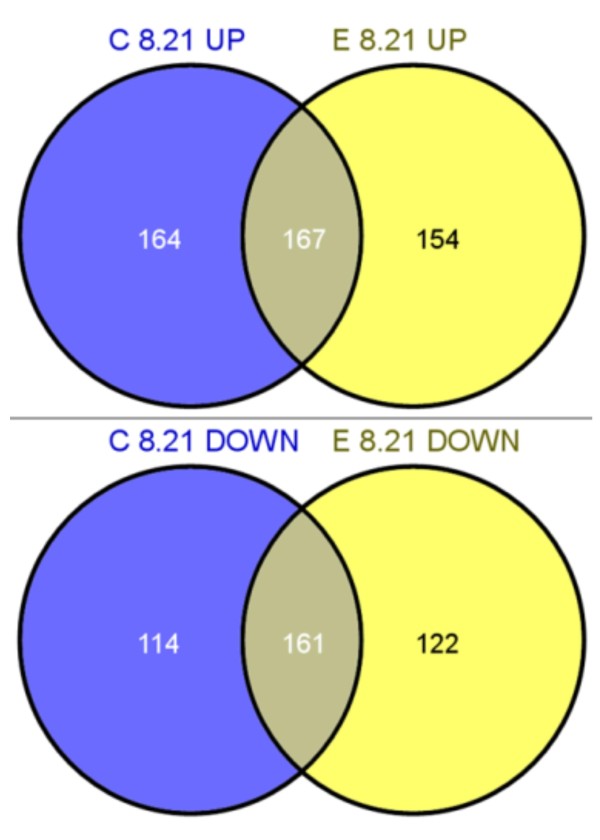
**Venn diagram**. Venn diagram illustrating the relationship of up- and downregulated annotated genes (*p *≤ 0.05, no log2 fold change) in the control (C) and expressing (E) strain in the pairwise comparison (8.21) of hypoxic (8) to normoxic (21) conditions. Up- or downregulation always refers to the lower oxygen setpoint, i.e. in this case to hypoxia. The intersections reflect equally regulated genes among both data sets.

As illustrated in the Venn diagrams (Figure 1), only around fifty percent of these genes were identically regulated in both the Fab-expressing and the control strain, indicating a different behaviour under hypoxic conditions. A direct comparison of the gene regulation pattern between expressing and control strain at the lowest oxygen concentration (8%), in contrast, revealed only six genes (see Table 3) to be differently expressed (adjusted *p*-value ≤ 0.05). For all of these six genes the log2 fold change was also greater than 0.59. Four of them showed higher transcript levels in the Fab producing strain, specifically, genes involved in a non-classical protein export pathway (*NCE103*), glycolysis (*PFK3*), the ergosterol pathway (*ERG25*) and in multidrug transport (*AQR1*). On the other hand, a high-affinity cysteine-specific transporter (*YCT1*) and a plasma membrane transporter of the major facilitator super family (*FLR1*) were significantly downregulated in the producing strain. In general, these data point at a major impact of oxygen rather than a consequence of heterologous protein production on the global transcriptional response of *P. pastoris*.

**Table 3:** Strain-dependent gene regulation

Gene Name	*p*-value	log2 fold change	Description
**E/C 8 up**			

NCE103	2.39E-02	1.15	*Carbonic anhydrase, involved in a non-classical protein export pathway*

PFK3	1.06E-02	1.34	*Pichia pastoris 6-phosphofructokinase gamma-subunit*

ERG25	3.29E-03	1.48	*Required in the ergosterol biosynthesis pathway*

AQR1	3.99E-02	1.67	*Multidrug transporter of the major facilitator superfamily*

**E/C 8 down**			

YCT1	3.20E-04	- 3.21	*High-affinity cysteine-specific transporter*

*FLR1*	4.08E-02	- 1.15	*Plasma membrane multidrug transporter*

List of differently regulated genes (*p*-value ≤ 0.05 and a log2 fold change ≥ 0.59) in both strains under hypoxic (8) conditions. E/C 8 up corresponds to the upregulated genes in the expressing strain (E) as compared to the control strain (C), while the downregulated genes are referred to as E/C 8 down.

**Figure 2:**
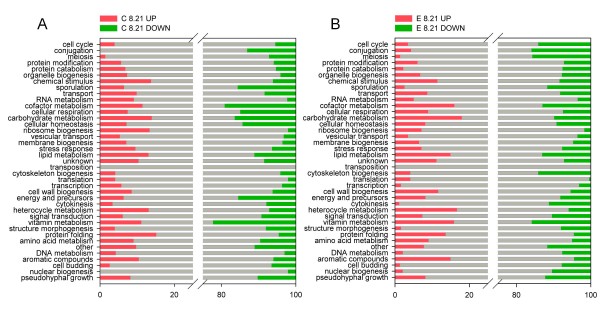
**Percentage distribution of regulated genes (cut-off *p *≤ 0.05) to their respective GO biological process term(s)**. This graph provides an overview of the GO groups that strongly responded to hypoxia. The percentage of upregulated (red), downregulated (green) and unregulated genes (white) were classified into their GO functional group(s) and illustrated as relative numbers summing up 100%. A: control strain (C), B: expressing strain (E); 8.21 = pairwise comparison of hypoxic (8) and normoxic (21) conditions, with the type of regulation (UP or DOWN) referring to the hypoxic setpoint

To gain an overview of the functional processes that are significantly correlated with a change in oxygen availability, we assigned all regulated genes that passed the *p*-value threshold to their respective GO functional group(s) (Gene Ontology based on *Saccharomyces *Genome Database SGD). The percentage distribution of the genes in each category is shown in Figure 2A and 2B. The most prominent biological processes that were exclusively induced under hypoxic conditions are *chemical stimulus*, *cell wall biogenesis*, *heterocycle metabolism*, *protein folding *and *cellular aromatic compounds*. The regulated genes in the GO groups *cofactor- and carbohydrate metabolic process*, *lipid metabolic process *and *vitamin metabolic process *showed very similar profiles of up and downregulation at the same time. On the other hand, hypoxia decreased the activity of genes involved in *conjugation, sporulation *and *pseudohyphal growth*.

The global tendencies of regulated biological processes overlap quite concordantly in both strains, with some exceptions: interestingly, the number of upregulated genes in the GO groups *RNA metabolism*, *protein catabolism*, *ribosome biogenesis*, *transcription *and *DNA metabolism *was higher in the control strain, while processes like *cellular respiration*, *carbohydrate metabolism*, *cellular homeostasis and amino acid metabolism *were more strongly enriched in downregulated genes. On the other hand, four GO categories appeared to comprise more downregulated genes in the producing strain, namely *cell cycle*, *meiosis*, *cytoskeleton biogenesis *and *nuclear biogenesis*.

It has to be emphasized that these results were obtained just employing the *p*-value threshold, excluding the log2 fold change threshold, to provide a more comprehensive transcriptional profile including also low but significant gene regulation. Thereafter, two selected GO groups, namely stress response and lipid metabolism, were analyzed in more detail in such a way that all the genes that were significantly up- or downregulated under hypoxia by at least 1.5-fold (log2 fold change ≥ 0.59) were categorized according to their functions (as discussed in section 'Other key cellular processes with important transcriptional changes'). The selection of these groups was either based on major differences between oxygen supply conditions or on the potential impact on protein production, as some of them appeared to be generally stronger regulated in the Fab producing strain.

The microarray data were validated by quantitative real-time PCR (qRT-PCR) including 1 reference gene and 14 target genes, which we selected according to their relevance for this study. Six of these genes were differently expressed between control and expressing strain under hypoxic conditions. We further selected six genes from the central carbon metabolism and compared their transcript levels between normoxic and hypoxic conditions in the expressing strain. qRT-PCR experiments also considered the genes encoding the light and the heavy chain of the Fab fragment, which were also on the DNA microarrays. From the expression data, the Fab heavy chain seemed completely unregulated when comparing the Fab producing strain versus the non producing strain. As suitable reference genes we chose β-actin (*ACT1*). The results are highlighted in Table 4 and indicate a good correlation between microarray and qRT-PCR data. In case of the Fab heavy chain we were able to demonstrate a 1.6 fold change when comparing mRNA levels between hypoxic and normoxic samples, which is similar to the fold change of 2.07 (log2 fold change of 1.05) as seen for the light chain (see Table 4). The higher Fab transcripts under control of the GAP (TDH3) promoter were also coherent with a transcriptional upregulation of glycolytic genes (and, particularly, of *TDH3*), as discussed later in section 'Transcriptional regulation of metabolic enzymes and fluxes', and positively correlated with the observed increase in the specific Fab titre.

**Table 4:** Correlation between microarray ad qRT-PCR data

**Target Gene**	**PIPA ORF**	**8/21 E array**	***p*-value**	**8/21 E qPCR**	**stdev**
			
*MDH1*	PIPA02244	-1.61	4.90E-03	-2.03	± 0.212
*FUM1*	PIPA02844	-2.07	2.23E-02	-1.42	± 0.121
*YDL124W*	PIPA01263	2.58	3.00E-04	1.52	± 0.139
*RKI1*	PIPA02895	2.44	2.50E-03	1.93	± 0.091
*CDC19*	PIPA00751	5.24	8.24E-10	3.71	± 0.189
*TDH3*	PIPA02510	4.34	5.44E-06	2.03	± 0.151
*Fab Hc*	-	1.65	8.80E-02	1.68	± 0.064
*Fab Lc*	-	2.07	8.30E-05	1.80	± 0.019
			
		**E/C 8 array**	***p*-value**	**E/C 8 qPCR**	**stdev**
			
*NCE103*	PIPA03864	2.21	2.30E-02	1.71	± 0.087
*PFK3*	PIPA09969	2.53	1.20E-02	3.21	± 0.128
*ERG25*	PIPA00945	2.79	3.00E-03	2.21	± 0.093
*AQR1*	PIPA04502	3.18	3.90E-02	4.12	± 0.188
*YCT1*	PIPA00376	-9.19	3.90E-04	-5.03	± 0.322
*FLR1*	PIPA02458	-2.22	4.10E-02	-1.78	± 0.161
*Fab Hc*	-	2.96	1.75E-01	10.31	± 0.397

Quantitative real-time PCR results compared with microarray data. Standard deviations (stdev) derive from triplicate measurements; all numbers reflect relative gene expression to the reference gene actin (*ACT1*). PIPA ORF = *Pichia pastoris *Open Reading Frame (http://www.pichiagenome.org); 8/21 E = pairwise comparison of hypoxic (8) and normoxic (21) conditions in the expressing strain (E); E/C 8 = pairwise comparison of expressing and control (C) strain under hypoxic conditions

### The effect of hypoxia on the proteome of *Pichia pastoris*

**Figure 3:**
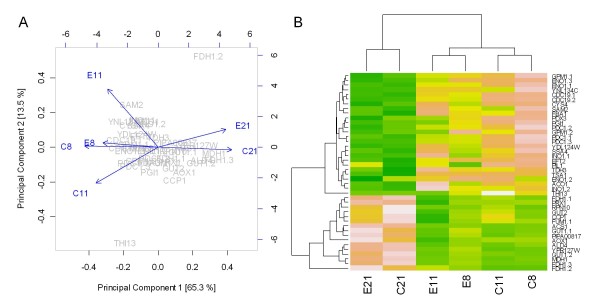
**Principal component analysis (PCA) and heat map of proteome data**. A: Principal component analysis of the proteome data in a 2D graph of PC1 and PC2. The biplot shows proteome data (scores) as labelled dots and treatment effect (loadings) as vectors for the expressing (E) and control (C) strain) at different oxygen concentrations in the inlet gas (21, 11 or 8% O2). Vectors that are close together are highly correlated in terms of the observed proteome for each treatment while vectors that are orthogonal are poorly correlated. PC1 correlates well with the change in oxygen conditions as the projection of the tips of the arrows on PC1 axis indicate. The effect of the 8% conditions appears stronger than the effect of 11% and both effects clearly differ from the 21% reference on each strain as indicated by the projections. B: Heat map presentation of a hierarchical cluster of the 45 proteins that show significantly different (*p *≤ 0.05) relative abundances in both strains (expressing (E) and control (C) strain) at different oxygen concentrations in the inlet gas (21, 11 or 8). The green colour represents low and pink colour represents high expression levels.

In parallel to the microarray experiments, we employed 2D-DIGE gels to measure the relative protein abundance changes at different levels of oxygen availability. To assure accuracy of the measurements and unbiased data, the protein extracts were labelled with both Cy3 and Cy5 fluorescent dyes and assigned the samples randomly to the protein gels according to the scheme in Additional file 1. We detected 85 spots with a significantly (1-way ANOVA *p*-value ≤ 0.05) different abundance pattern comparing the expression under normoxic and hypoxic conditions, and a smaller number of spots when comparing the proximate oxygen set points with each other (normoxic vs. oxygen-limiting, oxygen-limiting vs. hypoxic). A total of 45 spots could be excised from Coomassie Brilliant Blue stained protein gels and identified by LC-ESI-QTOF Tandem MS (see Additional file 2 for a representative 2D gel image and Additional file 3 for the list of identified protein spots). Some proteins showed more than one spot on the 2D gels indicating the existence of isoforms which probably derive from posttranslational modification (PTM) events such as phosphorylation, glycosylation or limited proteolysis (see, for example [19]). In order to obtain a simplified structure of the behaviour of all identified proteins under different conditions of oxygen availability, we subjected the relative protein abundances (see Materials and Methods) to principal component analysis (PCA) and heat map clustering. PCA projection demonstrates that the maximum variability in the dataset occurs between oxygen set points 21% and 8% (see Figure 3A) with the first component covering 65.8% of the data variance. This result is also reflected in the heat map, where two major clusters separating the protein abundance profile at normal oxygen levels from that at limiting and hypoxic levels can be observed (see Figure 3B). All the identified proteins exhibited a similar expression profile when comparing the Fab producing strain and the non producing control strain.

Most of the proteins with a high abundance at low oxygen are involved in *glycolysis*, *amino acid metabolism *and *general stress response*. Identified proteins with a low expression in hypoxic conditions mostly belong to the functional processes *TCA cycle*, *vitamin metabolism *and *oxidative stress response*.

### Multi-level analysis of the *P. pastoris *central carbon metabolism adaptation to hypoxia

The impact of reduced oxygen supply on the core metabolism was readily observed, both in the biomass yields and the profile of secreted by-products such as ethanol and arabitol, reflecting the adaptation from a respiratory to a respiro-fermentative metabolism (Table 1). Since all cultivations were carbon-limited (residual glucose concentration in the reactor under detecting concentrations), the decrease in the biomass yield under oxygen-limiting and hypoxic conditions resulted in an increase of specific glucose uptake rates under such conditions.

Biosynthetically directed fractional (BDF) ^13^C-labeling of proteinogenic amino acids combined with 2D-NMR enabled the analysis of metabolic flux ratios (METAFoR analysis). The metabolic flux ratios were calculated using the relative abundances (*f*-values) of intact carbon fragments arising from a single source molecule of glucose (Additional file 4). The calculated flux ratios are shown in Table 5. As expected, the *f*-values obtained for this series of cultivations confirm that the proteinogenic amino acids are primarily synthesized in *P. pastoris *according to the pathways documented for *S. cerevisiae*, as previously reported [20].

**Table 5:** Metabolic flux ratio (METAFoR) analysis results

% Fraction of total pool	Expressing Strain			Control Strain		
		
	**21% O**_**2**_	**11% O**_**2**_	**8% O**_**2**_	**21% O**_**2**_	**11% O**_**2**_	**8% O**_**2**_
**Pep from PPs, upper bound**	**50 ± 9**	**23 ± 6**	**15 ± 7**	**39 ± 9**	**32 ± 8**	**15 ± 6**
R5P from T3P and S7P (transketolase)	71 ± 2	78 ± 2	70 ± 2	66 ± 2	70 ± 2	62 ± 2
R5P from E4P (transaldolase)	44 ± 2	24 ± 2	23 ± 2	40 ± 2	29 ± 2	24 ± 2
Ser originating from Gly and C1-unit	61 ± 4	68 ± 4	68 ± 4	62 ± 4	69 ± 4	72 ± 4
Gly originating from CO_2 _and C1-unit	10 ± 4	13 ± 3	13 ± 3	6 ± 4	12 ± 3	10 ± 3
Pep originating from Oaa_cyt _(PepCK)	0 ± 4	0 ± 8	0 ± 10	2 ± 5	0 ± 10	5 ± 11
Oaa_mit _originating from Pep	44 ± 2	32 ± 2	44 ± 2	42 ± 2	35 ± 2	41 ± 2
**Oaa**_**mit **_**originating from Oaa**_**cyt**_	**n.a**.	**43 ± 3**	**55 ± 3**	**n.a**.	**44 ± 3**	**51 ± 3**
**Oaa**_**cyt **_**originating from Pep**	**n.a**.	**63 ± 3**	**64 ± 4**	**n.a**.	**66 ± 3**	**66 ± 4**
Oaa_cyt _reversibly converted to Fum	63 ± 11	7 ± 5	10 ± 5	63 ± 11	11 ± 4	9 ± 4
**Flux through malic enzyme, upper bound**	**1 ± 4**	**n.d**.	**n.d**.	**1 ± 6**	**n.d**.	**n.d**.
Flux through malic enzyme, lower bound	1 ± 2	n.d.	n.d.	0 ± 3	n.d.	n.d.

Origins of metabolic intermediates during growth of *P. pastoris *in glucose-limited chemostat ^13^C-labelled cultures at D = 0.1 h^-1 ^at different fractions of oxygen in the chemostat inlet gas. Values for both control and Fab producing strains are given. Ratios highlighted in bold have been used as constraints for metabolic flux analysis. n.a., not applicable; n.d., not determined. See main text for explanation.

**Figure 4:**
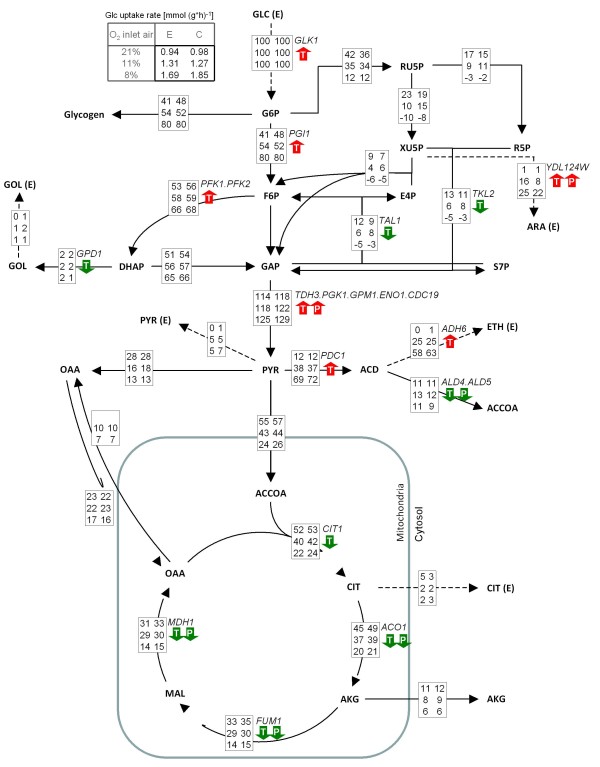
**Metabolic flux distributions in *P. pastoris *Fab-expressing and control strains under different oxygenation conditions**. Relative net flux distributions of *P. pastoris *X-33/pGAPαA_Fab and X-33/pGAPαA in glucose-limited chemostats at a D = 0.1 h^-1 ^under different oxygenation conditions. Fluxes are shown as relative fluxes normalized to the specific glucose uptake rate (expressed as mmol glucose g^-1 ^DCW h^-1^) in the corresponding experiment. The specific glucose uptake rates corresponding to the different oxygenation conditions and strains are given at the top of the figure. The fluxes for each reaction in the network corresponding to 21%, 11% and 8% oxygen in the bioreactor inlet gas are given from top to bottom; the flux values from the Fab-producing strain are shown on the left and those from the corresponding control strain on the right. The transport of Oaa across the mitochondrial membrane under normoxic conditions is given as a single net influx value. Fluxes with SD values are provided in the Additional file 5. Arrows indicate higher (red) or lower (green) mRNA levels (T) and protein abundances (P) during hypoxia compared to normoxia. The corresponding gene/protein names (in italics) are displayed above the arrows, while all metabolite names are indicated in bold letters: GLC = glucose; G6P = glucose-6-phosphate; F6P = fructose-6-phosphate; GAP = glyceraldehyde-3-phosphate; DHAP = dihydroxyacetone phosphate; GOL = glycerol; RU5P = ribulose-5-phosphate, XU5P = xylulose-5-phosphate; R5P = ribose-5-phosphate; ARA = arabitol; S7P = sedoheptulose-7-phosphate; PYR = pyruvate; ACD = acetaldehyde; ETH = ethanol; ACOOA = acetyl CoA; OAA = oxaloacetate; CIT = citrate; AKG = alpha-ketoglutarate; MAL = malate; (E) = external

In ^13^C-based metabolic flux analyses (^13^C-MFA), the metabolic flux ratios determined by METAFoR were used as additional constraints for the stoichiometric equation system to be able to solve the metabolic flux distribution without including cofactors (NADH, NADPH and ATP), O_2 _and CO_2 _in the metabolite mass balances. The net fluxes for the Fab-producing and control strains growing under different oxygenation conditions are shown in Figure 4. The net fluxes and their standard deviations are included in Additional file 5.

The most prominent feature, as already indicated by the METAFoR analysis (Table 5), was the similarity in flux estimates between corresponding Fab-producing and control strains datasets. Nevertheless, as expected, clear differences were observed when comparing flux patterns corresponding to different oxygenation set points. In general terms, the metabolic adaptation from oxidative towards respiro-fermentative growth was accompanied by complex changes of carbon flux throughout the whole central carbon metabolism, as previously described in other yeasts (*S. cerevisiae *[21,22], *P. anomala *[23,24]).

#### Glycolytic and Pentose Phosphate Pathway (PPP) fluxes

The METAFoR analysis showed that in fully aerobic conditions up to 50-39% of phosphoenolpyruvate (Pep) was originated from the pentose phosphate pool. In contrast, the fraction of Pep from the pentose phosphates assuming a maximal contribution of PPP was clearly lower under hypoxic conditions, only about 15%.

Moreover, the ^13^C-MFA results (shown in Figure 4) indicate that this decrease of the relative PPP flux was the result of both an increased glycolytic flux, and a decrease in the specific flux through the oxidative branch of the PPP. As previously observed in *S. cerevisiae *[22], the glycolytic flux increased progressively as the oxygen availability decreased.

As already inferred from the macroscopic data, fluxes through some fermentative pathways were increased upon adaptation from respirative to respiro-fermentative metabolism, particularly the fluxes towards the formation of ethanol and arabitol. Production of arabitol had a clear impact on the flux ratios and on the distribution of fluxes through the PPP: The fraction of pentose phosphates showing the reversible action of a transketolase reaction was generally high (> 60% in all cultivations), with no clear trend, whereas the fraction of pentose phosphates showing the reversible action of a transaldolase clearly decreased at low oxygen availability. Overall, the ^13^C-MFA results showed that under normoxic conditions there was an important net contribution of the PPP to glucose catabolism (flux of PPP intermediates to triose phosphates). In contrast, as oxygen availability was decreased and, particularly, when arabitol was produced, this contribution was clearly reduced or, even some of the reactions of the non-oxidative PPP branch showed inverted directionality under hypoxic conditions.

#### Fluxes around the pyruvate node, intercompartmental transport and Tricarboxylic Acids (TCA) cycle

Following the METAFoR analysis, distinct flux changes were observed for the different pathways utilizing pyruvate (Pyr), that are, pyruvate carboxylase (anaplerosis), pyruvate decarboxylase (fermentative pathways, pyruvate dehydrogenase by-pass), and pyruvate dehydrogenase (direct import of Pyr to the mitochondria). The relative anaplerotic flux (the anaplerotic flux ratio defined as the fraction of mitochondrial oxaloacetate Oaa_mit _molecules originating from Pep) was around 44-41% under normoxic and hypoxic conditions, while in oxygen-limiting conditions was slightly lower, 35-32%. Hence, pyruvate carboxylase catalyzed reaction seemed to be the major anaplerotic reaction under all conditions, and it was accompanied by a substantial transport of carbon (cytosolic oxaloacetate Oaa_cyt _and/or other TCA cycle intermediates) from cytosol to mitochondria. Nevertheless, an important relative carbon efflux from the mitochondria may be occurring, as only 66-63% of Oaa_cyt _appears to be directly synthesized from Pep under oxygen-limiting and hypoxic conditions. As previously observed for *P. pastoris *[20] and other yeasts (e.g. *S. cerevisiae *and *P. stipitis*, [22,25]), cells growing aerobically in glucose-limited chemostats show a bidirectional transport of Oaa and/or other TCA cycle intermediates across the mitochondrial membrane. However, calculation of flux ratios defining the fraction of Oaa_mit _from Oaa_cyt _and, Oaa_cyt _from Pep under normoxic conditions was not possible. The labelling patterns of cytosolic and mitochondrial Oaa pools are accessible through the observation of aspartate labelling patterns (shown to be synthesized from Oaa_cyt _in yeast in previous studies [26]) and glutamate (synthesized from mitochondrial 2-oxoglutarate, and therefore accessing to Oaa_mit _labelling patterns). Strikingly, the fraction of intact C2-C3 bonds of Oaa, which is often used to calculate these flux ratios, where approximately equal for Oaa_cyt _and Oaa_mit _(that is, the fraction of intact Cα-Cβ bonds in Asp/Thr and Glu were equal as revealed by the *f*-values of Asp, Thr and Glu, Additional file 4). This could be explained by an extremely fast exchange between cytosolic and mitochondrial pools of TCA cycle intermediates (near equilibrium), resulting in identical labelling patterns in the amino acids synthesized from such pools. However, this possibility can be excluded, as the Asp-Cβ, Thr-Cβ and Glu-Cα labelling patterns under normoxic conditions were not identical (Additional file 4). Notably, the reversibility of the interconversion of cytosolic Oaa to other cytosolic TCA cycle intermediates (defined here as the Oaa_cyt _interconversion to fumarate ratio) was clearly higher under normoxic conditions, suggesting that aspartate, Oaa, and malate might be participating in a redox shuttle (e.g. malate-Asp and/or malate-Oaa shuttles) for translocation of NADH across the mitochondrial membrane, as described by Bakker et al [27].

It is worth noting that, although the flux through the pyruvate dehydrogenase (PDH) bypass was not considered in our metabolic model (see Materials and Methods for explanation), its activity should not be totally excluded since Crabtree negative yeasts are reported to have activity on this pathway [28]: In contrast to *S. cerevisiae*, which does not synthesise carnitine *de novo *(essential for carnitine acetyltransferase-mediated transport of cytosolic acetyl-CoA (AcCoA) to the mitochondria [29]), a complete carnitine biosynthesis pathway has been characterized in *Candida albicans*, and the corresponding 4 genes have been identified [30]. Interestingly, the *P. pastoris *genome contains putative homologues to these genes [16,31]. Moreover, it should be mentioned that we observed a strong downregulation of the mitochondrial acetyl-CoA synthetase Acs1p on the mRNA (log2 fold change = -4.1) and protein level (average ratio = -2.1) when comparing hypoxic with normoxic conditions. Acs1p is essential for the contribution of mitochondrial PDH bypass to the formation of mitochondrial acetyl-CoA. Also, a significant downregulation in expression levels of *CAT2 *and *YAT2 *homologues involved in carnitine transport to mitochondria was observed.

**Figure 5:**
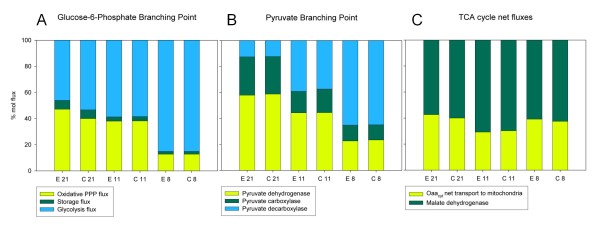
**Fractional distributions of carbon fluxes in metabolic branching points derived from ^13^C-MFA**. Fractional distributions of carbon fluxes. A: the glucose-6-P flux split to glycolysis and PPP B: the pyruvate branching point, and C: the TCA cycle, in *P. pastoris *Fab-producing (E) and control (C) strains growing in glucose-limited chemostats at D = 0.1 h^-1^, in 21%, 11% and 8% oxygen in the chemostat inlet gas.

Overall, carbon flux distributions at the pyruvate branching point (see Figure 5) clearly show the shift from respiratory to respiro-fermentative metabolism: fluxes through the pyruvate dehydrogenase pathway decreased when decreasing oxygen availability, whereas the flux through the pyruvate decarboxylase pathway increased, reflecting the production of ethanol. Also, the anaplerotic flux though the pyruvate carboxylase pathway drastically decreased when oxygen availability was reduced: Under fully aerobic conditions the fraction of carbon flux to the TCA cycle through this pathway was about 29% (calculated only as a net transfer of Oaa_cyt _across the mitochondrial membrane), whereas under oxygen-limiting and hypoxic conditions exchange fluxes decreased, resulting in lower net carbon fluxes into the TCA cycle (16-18% and 12% in oxygen-limiting and hypoxic conditions, respectively).

Remarkably, although variations in the carbon flux distribution around the pyruvate branch and TCA cycle activity were observed, mitochondrial transporters such as *DIC1*, *OAC1*, *SFC1 *showed no significant change on the transcriptional level. Only *YIA6*, which is involved in NAD^+ ^transport into the mitochondria (and has a disputed role as Pyr transporter), was downregulated under hypoxic conditions. As already mentioned before, the carnitine transporters *CAT2 *and *YAT2 *were also downregulated under such conditions.

Limitation in oxygen availability reduced the respirative net carbon flux through the TCA cycle (that is, the net flux of α-ketoglutarate through the TCA cycle to Oaa_mit_, Figure 4). Nevertheless, the fraction of the net carbon flux in the TCA cycle corresponding to the respirative carbon flux from α-ketoglutarate (or relative TCA cycle activity) remained between 60-70%, that is to say, the relative anaplerotic fluxes were between 30-40%. Similar results have been previously observed in *S. cerevisiae *under aerobic conditions [22]. No significant contribution xzfor malic enzyme flux could be observed (Table 5), so the pyruvate carboxylase pathway was the only anaplerotic supply to the TCA cycle.

### Transcriptional regulation of metabolic enzymes and fluxes

A good qualitative correlation between both transcriptional and proteomic levels and the corresponding *in vivo *fluxes in the different oxygenation conditions was observed for glycolysis, fermentative pathways and TCA cycle (Figure 4). Exceptions were *ZWF1 *and *GND2*, coding for enzymes involved in the oxidative branch of the PPP, which did not appear to be significantly regulated, in spite of the strong variation observed in the metabolic fluxes through this pathway. This was also the case for the *GPD1 *gene, coding for the enzyme involved in glycerol formation, which was significantly downregulated at the transcript levels, whereas the flux to glycerol excretion was very low or even zero under all oxygenation conditions.

Following proteomic analyses, the glycolytic enzymes Pgi1p, Fba1p, Tdh3p, Gpm1p, Eno1p and Cdc19p were identified to be strongly induced, while TCA cycle proteins Aco1p, Fum1p and Mdh1p show very low abundance in hypoxic conditions, pointing at a redirection of the central carbon metabolism towards fermentation. These results were expected and are consistent with previously obtained data on yeast physiology under oxygen deprivation, where glycolytic activity in anaerobically grown *S. cerevisiae *was shown to be higher than in aerobiosis on the proteome level [32,33]. These studies, however, revealed a weak correlation between transcriptome and proteome data of this key cellular process in *S. cerevisiae*, which is in strong contrast to our results where the transcript levels indeed correlate with the proteome profile and *in vivo *fluxes of the central carbon metabolism.

Notably, transcriptional levels of *TDH3 *under hypoxic conditions were 2.7 fold higher in the Fab producing strain than in the control strain. At the protein level, however, the average ratios were quite similar with 1.5 for the expressing and 2.1 for the control strain. *TDH3 *codes for glyceraldehyde-3-P dehydrogenase, an enzyme playing an integral role in glycolysis. Interestingly, Tdh3p has also been recognized to be involved in the initiation of apoptosis in *S. cerevisiae *[34]. Furthermore, it has been also found in the yeast cell wall [35], suggesting that *TDH3 *may indeed encode a multifunctional protein.

Along with an adaptation towards a fermentative metabolism cells have to remove excess redox equivalents that accumulate during biomass synthesis and excretion of oxidized metabolites [36]. Anaerobically grown *S. cerevisiae *produces glycerol in order to reoxidize accumulated NADH during amino acid biosynthesis [37]. In contrast, we have recently described that hypoxically growing *P. pastoris *cells secrete arabitol, a 5-carbon sugar alcohol, but not glycerol [18]. Arabitol (and glycerol) accumulation has been previously observed in *Pichia anomala *cultures during growth in high-salt environments and on highly concentrated sugar substrates [38,39]. Dragosits and co-workers recently demonstrated intracellular arabitol accumulation in *P. pastoris *chemostat cultivations grown under conditions of high osmolarity [17]. In fact, it has been suggested that arabitol has the same physiological role as glycerol in the protection to osmotic stress [40]. Similarly, arabitol might also be involved in maintaining the redox balance during fermentative growth.

There is no identified protein in our data clearly assigned to arabitol biosynthesis. D-arabitol 2-dehydrogenase, the only protein that is described to form arabitol from ribulose in *P. pastoris*, could not be detected in our protein gels. However, we speculate that Ydl124wp, identified as a putative NADPH-dependent alpha-keto amide reductase and described to have similarity with Gre3p, the major aldose reductase in *S. cerevisiae *[41,42], may be involved in the formation of the 5-carbon sugar alcohol. Since D-ribulose and D-xylulose from the pentose pathway are the main precursors for the formation of arabitol in many fungi [43,44] it may be assumed that Ydl124w is involved in these reductive activities. The enzyme levels of this protein were significantly induced under hypoxic conditions, with an average fold change of 1.6 and 2.3 for the control and expressing strain, respectively. At the mRNA level this upregulation was even more apparent with log2 ratios of 1.5 and 1.9.

Gut2p, a mitochondrial enzyme that is associated with redox balance maintenance via the glycerophosphate shuttle under aerobic conditions, showed significantly decreased abundance at the protein (average ratio below -1.5) as well as mRNA level (log2 fold change ≤ -1.54) under hypoxia, as it is not needed under this condition to transfer reducing equivalents to the respiratory chain. The same result was obtained for Gut1p, which together with Gut2p is responsible for glycerol degradation. Fdh1p, a NAD^+^-dependent formate dehydrogenase, is also downregulated when oxygen is scarce. Singh and co-workers described a possible relation between pyruvate break-down and the production of formate [45]. Since the pyruvate pathway is directed towards ethanol formation under hypoxic conditions, the synthesis of formate might be reduced and could explain the lower abundance of formate dehydrogenase.

Although the methanol pathway is tightly repressed under growth on glucose, the basic level of Aox1p was strictly downregulated under hypoxic conditions as has been shown previously in glucose-limited conditions [7,17]. A plausible explanation for the low abundance of Aox1p is the higher glycolytic activity under low oxygen conditions and the repressive effect of glucose on *AOX1 *transcription. This effect could also be responsible for the weak expression of the proteins Acs1p, an acetyl-CoA-synthetase, and Ald4p, a mitochondrial aldehyde dehydrogenase, whose expression is also glucose repressed.

### Other key cellular processes with important transcriptional changes

#### Lipid metabolism and membrane biogenesis

**Figure 6:**
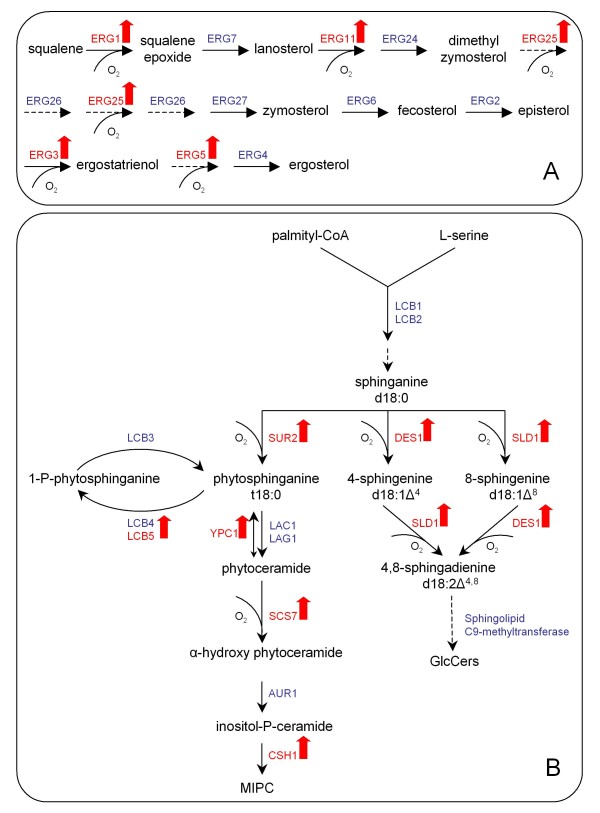
**Scheme of pathways involved in lipid metabolism**. Schematic overview of the discussed pathways involved in lipid metabolism. Colour code: red = upregulated genes; green = downregulated genes; and blue = un-regulated genes under hypoxic conditions (log2 fold change ≥ 0.59 and *p *≤ 0.05) A: Sphingolipid metabolism in the yeast *P. pastoris *adapted from [51]. MIPC = mannosyl-inositol-phosphorylceramide, GlcCer = glucosylceramides B: Outline of the post-squalene ergosterol biosynthetic pathway, dashed arrows indicate no specification of intermediates

Lipid metabolism has successively become a focus of attention for linking many important pathways to its intermediate substrates. Sterols for example, long time relegated as passive metabolites modulating the membrane structure, play an important role as primary sensors of environmental stresses by adjusting the fluidity of the plasma membrane to such perturbations [46,47]. The cellular lipid metabolism is highly susceptible to hypoxia since the biosynthesis routes for the most essential lipids - ergosterol and fatty acids - require molecular oxygen. Anaerobically grown *S. cerevisiae *has an impaired ability to produce ergosterol, which consequently has to be added to the medium together with Tween80 as a source for unsaturated fatty acids [48,49]. In this study the medium was not supplemented with either of these reagents since our chemostat cultures were run under severe oxygen limitation but not under strictly anaerobic conditions. As a consequence and due to the lack of exogenous ergosterol uptake, we observed increased transcript levels for a number of enzymes that catalyze oxygen-consuming reactions of the ergosterol pathway (*ERG1, ERG3, ERG5, ERG11 *and *ERG25*) which may be upregulated upon hypoxia for compensation of intermediate substrate deficit (see Figure 6A). *NCP1*, a cytochrome P450 reductase that is reported to be uniformly regulated with the key enzyme *ERG11*, also demonstrated increased mRNA levels under hypoxia. It has to be stated that the transcription of *ERG25 *was not only induced by oxygen scarcity but also under recombinant protein-producing conditions.

Besides ergosterol as one of the major plasma membrane components in yeast, sphingolipids represent another class of lipids with considerable importance, since they interact with ergosterol to form small platforms ("rafts") in the cell membrane. Along with the changes in the transcription pattern of the ergosterol pathway, we also observed a hypoxic induction of four sphingolipid synthesis genes (*SUR2, SCS7, DES1*, and *SLD1*) under hypoxic conditions (see Figure 6B for a schematic overview). Interestingly, similar as demonstrated for ergosterol, all of these enzymes need molecular oxygen as substrate, unlike others whose mRNA levels remained constant (*LAC1, LAG1*, sphingolipid C9-methyltransferase). *SLD1 *(Δ8 sphingolipid desaturase), *DES1 *(Δ4 sphingolipid desaturase) and the C9-methyltransferase were recently identified and characterized by Ternes and co-workers to be responsible for the synthesis of glucosylceramides (GlcCer) in *P. pastoris *[50]. In a similar study, a *P. pastoris *mutant strain deficient in the endogenous *DES1 *gene was not able to produce glucosylceramides either [51]. This is in strong contrast to *S. cerevisiae*, who lacks this common class of sphingolipids.

*LAC1 *and *LAG1 *are the only ceramide synthases in *P. pastoris*. If the cells produced more ceramides (forming the backbone of sphingolipids) at low oxygen levels, at least one of these two genes would be upregulated. Since this is not the case, we speculate that the cell activates the oxygen-dependent enzymes in order to sustain the sphingolipid metabolism and growth in spite of oxygen scarcity.

*YPC1*, a ceramidase, might not fit in this picture since it also has a reverse, but minor ceramide synthase activity. Its mRNA level was increased to a similar extent (2.2-fold) as *LCB5*, responsible for the phosphorylation of long chain bases in a side-branch reaction of the sphingolipid metabolism. Also *CSH1*, a probable catalytic subunit of a mannosylinositol phosphorylceramide (MIPC) synthase, was upregulated under low oxygen. However, the genes encoding the subunits of serine-palmitoyltransferase (*LCB1, LCB2*), the rate-determining step of sphingolipid synthesis in *S. cerevisiae*, were not induced, and if we exclude any posttranscriptional regulations, this hypothesis about the upregulation of oxygen-consuming reactions might be true.

A mentionable induction upon hypoxic growth was further noted for the genes *PDR16 *(2.8-fold), *YPL206C/PGC1 *(3.5-fold) and *PDX3 *(5-fold). Pdr16p is a phosphatidylinositol transfer protein of the Sec14p family (involved in protein secretion) and was attributed a role in altering the lipid composition of the plasma membrane [52]. *PGC1 *regulates the phosphatidylglycerol (PG) content by degradation of PG to diacylglycerol (DAG) via a phospholipase C activity, as recently reported [53]. *PDX3 *is closely related with sterol, fatty acid and cytochrome content in yeast cells [54]. The gene product Pdx3p, a pyridoxamine phosphate oxidase, was additionally shown to have a significantly higher abundance at low oxygen. A similar positive correlation between proteome and transcriptome data for enzymes involved in lipid metabolism was observed for Sam2p, an S-adenosylmethionine synthetase with a broad range of biological functions. Besides its role in the methylation of proteins and lipids, S-adenosylmethionine (AdoMet) was further attributed a role in the synthesis of phospholipids [55], which is also the case for Ino1p, an inositol 1-phosphate synthase.

*OLE1*, a hypoxic gene encoding a key enzyme (Δ-9 fatty acid desaturase) in the synthesis of unsaturated fatty acids, was also significantly expressed under hypoxic conditions. Along with this result, the breakdown of fatty acids was highly impaired by the strong downregulation of the oxygen-dependent β-oxidation pathway (*FAA1, FAA2, POX1, ECI1, FOX2, POT1 *and *SPS19*), and the genes required for peroxisomal division and metabolite transport, *PEX11 *and *ANT1*, respectively.

Since alterations in the sterol-sphingolipid balance not only result in defects in the physical properties of membranes, but also affect strongly related events like cell signalling [56] or secretory transport to the cell surface [57,58], we speculate that this imbalance might somehow also influence recombinant protein secretion. Sphingolipids or sterols, similar to secretory proteins, are first synthesized in the ER and then further processed in the Golgi apparatus, where they are targeted for the proper distribution to the cell surface. In the trans-Golgi network, where secretory vesicles segregate to exchange cargo proteins and lipids between membrane-bound organelles [59,60], lipid rafts play a pivotal role as sorting point [61]. Pma1 for example, an H^+^-ATPase and abundant plasma membrane protein, is misrouted to the vacuole in a mutant strain of *S. cerevisiae *with an inability in sphingolipid acyl chain elongation [62]. Gap1, a general amino acid permease, meets a similar fate in the absence of sphingolipid neosynthesis and is also sorted to the vacuole instead of the plasma membrane [63]. The heterologous Fab is a soluble protein and probably not directly affected by alterations in the sphingolipid metabolism as it is not targeted to the plasma membrane, however, the degradation of other integral membrane proteins might favour recombinant protein secretion.

Recent experiments in our lab (data not shown) demonstrated that Tween80, a non-ionic surfactant, stimulated the production of the recombinant extracellular Fab fragment considerably. A similar result was also obtained by Apte-Deshpande and co-workers [64] for a recombinant *P. pastoris *strain producing a human growth hormone upon methanol induction. This stimulating effect of surfactants has further been observed in other host organisms including filamentous fungi and bacteria, where authors speculate about a possible correlation with (1) changes in the electrochemical membrane gradients by an altered Na^+^/K^+ ^ratio [65] and (2) an altered membrane stability, even in transport vesicles, leading to enhanced membrane fusion [66]. These explanations would support our hypothesis about a contribution of membrane properties to improved protein secretion.

#### Stress response

In this study, *P. pastoris *was exposed to severe oxygen limitation as environmental stress factor. As a consequence and considering also the additional burden of heterologous protein production, the cellular stress responses were highly activated under hypoxic growth conditions. At the protein level we identified 3 stress-associated proteins that were significantly induced upon a shift to hypoxic conditions, namely Pil1p, Tsa1p and Ssa4p. While Pil1p is a component of the eisosomes that mark endocytic sites in the plasma membrane, Tsa1p and Ssa4p are molecular chaperones. On the other hand, Rpn10p, a regulatory particle of the proteasome which is involved in the degradation of ubiquitinated proteins, had a lower abundance under hypoxic conditions. The same trend was demonstrated for 3 further stress-related proteins: Ccp1p (cytochrome-c peroxidise) and Prx1p (thioredoxin peroxidase) are mitochondrial proteins and involved in oxidative stress responses, while Ypr127wp is an uncharacterized protein that shows similarity to the *Schizosaccharomyces pombe *pyridoxal reductase [67].

At the transcript level there was a considerable upregulation of genes involved in oxidative (*SVF1, UBA4, AHP1, TSA1, GAD1, NCE103 *and *OXR1*) and osmotic stress responses (*AGP2, RRD1, PBS2 *and *CAB3*), and of those encoding molecular chaperones (*HSP104, HSP42, HSP31, ZPR1, LHS1*, and genes from the Hsp40/DnaJ family: *XDJ1, SIS1 *and *SCJ1*). Many chaperones are important assistants during the protein folding process and provide for a quality checkpoint so that only correctly folded polypeptides are released into the secretory pathway. *TSA1*, one of the genes with the strongest overexpression (almost 6-fold) and with an increased abundance at the proteome level under hypoxia (see above), is a peroxidase under normal conditions and only shifts to its chaperone function in response to stress [68]. It belongs to the so-called 'moonlighting proteins' that have multiple functions, which can vary as a consequence of changes in their proximate environment. *TSA1 *functions as antioxidant on actively translating ribosomes and thereby maintains the integrity of the translation apparatus. But it was also shown to suppress thermal aggregation by binding to unfolded proteins. Stresses and other events that disrupt/overload the ER folding mechanism can cause accumulation of such unprocessed polypeptides and provoke the unfolded protein response (UPR) (reviewed in [69]). It was recently shown that antibody heavy chain fragments (which may remain partly unfolded) were retained within organelles of the secretory pathway in a recombinant *P. pastoris *strain, indicating a major bottleneck in the secretion process [70]. In the same study, overexpression of heterologous *HAC1*, the transcriptional activator of UPR in *S. cerevisiae*, clearly induced the UPR-regulated genes *KAR2*/BiP and *PDI1*. While the mRNA levels of *HAC1 *and *PDI1 *were significantly increased by hypoxia in our work, the transcription of *KAR2 *was not affected. However, we observed a significant induction of *LHS1*, a co-chaperone of *KAR2 *and likely to be the *KAR2 *nucleotide exchange factor. It is possible that the *S. cerevisiae*-derived *HAC1 *exerts a slightly different function in *P. pastoris *than the homologous one by regulating other UPR related genes [71]. In this context, also *IRE1*, a gene that senses misfolded proteins in the ER through interaction with *KAR2 *and activation of *HAC1 *was not induced. Interestingly, UPR was also reported to be triggered upon lipid deprivation in order to coordinate membrane synthesis in *S. cerevisiae*, in which *HAC1 *plays a role in mediating phospholipid biosynthesis [72]. This finding could be reasonably linked to the observed changes in the lipid balance during oxygen scarcity and may also explain a different regulation of UPR related genes.

*ERO1*, which is well-known for its crucial role in protein disulfide bond formation and redox homeostasis in the ER, was strongly activated under severe hypoxia (6.5-fold). It interacts with PDI to initiate the transfer of oxidizing equivalents to folding proteins [73]. In the presence of oxygen, *ERO1 *generates H_2_O_2 _while in conditions of severe hypoxia other compounds (i.e. FAD) serve as electron acceptors for *ERO1 *thus reducing the accumulation of reactive oxygen species in cells with heavy loads of protein thiols in their secretory pathway [74].

The overexpression of the UPR genes *PDI1*, *ERO1 *and *HAC1 *were previously reported as helper factors for enhanced recombinant protein secretion [2], which provides the presumption that their heavy induction upon hypoxia could benefit protein expression in a similar way or even stronger by a more synergistic effect. Another gene attracting attention due to its involvement in the translocation of soluble secretory proteins and insertion of membrane proteins into the ER membrane was *WSC4*, encoding a cell wall integrity and stress response component with a transmembrane receptor activity. Unlike in a study by Kimata and co-workers [75] where *WSC4 *was mentioned to be downregulated by UPR in *S. cerevisiae*, it was significantly induced under low oxygen conditions in our study, thus indicating a form of induction other than unfolded proteins.

It is also worthwhile mentioning that the stress related genes *NCE103*, a carbonic anhydrase, and *PFK3*, encoding a gamma subunit of the 6-phosphofructokinase complex, were considerably upregulated not only in hypoxia but also in the Fab producing strain, pointing at a certain protein-expression related regulation. PFK3 was recently reported as a novel form of the hetero-oligomeric enzyme 6-phosphofructokinase in *P. pastoris*, but with no similarity to classic PFK subunits [76]. In the same study it was shown that the gamma-subunit tightly regulates the glucose metabolism by fine-tuning PFK activity via AMP and ATP, providing a rapid adaptation to perturbations in the energy balance during environmental changes, like in the case of hypoxia. The enhanced transcript levels in the Fab-expressing strain can be explained by the additional energy demand during protein producing conditions.

*NCE103 *was regulated similarly to *PFK3 *in response to oxygen-limiting and protein-producing conditions. *NCE103 *was described by Cleves et al. [77] to encode for a protein that is a substrate of a non-conventional secretion pathway in yeasts. In a later study a *S. cerevisiae *strain deleted in *NCE103 *showed a growth-defect phenotype in the presence of oxygen and enhanced sensitivity to H_2_O_2_, thus suggesting a protective function against by-products of cellular respiration [78]. This contradicts our results where *NCE103 *was highly induced under hypoxic conditions. Clark and co-workers [79] also questioned the proposed antioxidant activity of *NCE103 *and detected a functional carbonic anhydrase activity instead. They even suggested a correlation between *NCE103 *activity and the supply of sufficient bicarbonate for lipid biosynthetic processes. This hypothesis might explain the higher *NCE103 *activity under oxygen scarcity. Its elevated induction in the protein producing strain could be explained by the anti-oxidative effect on ER-oxidative stress derived from UPR. More studies will be necessary to elucidate the proposed function in non conventional protein export.

## Conclusions

In this study we demonstrated that a systems biology approach integrating 2D DIGE proteome, DNA microarrays and metabolic flux analyses, can serve as a valuable tool to investigate the impact of key environmental parameters on host cell physiology under recombinant protein-producing conditions. Furthermore, the use of chemostat cultures allowed for well-controlled and reproducible culture conditions, essential for acquiring reliable data sets from different analysis levels. Such an approach becomes particularly attractive when the given conditions are known to have beneficial effects on protein secretion, as in the case of reduced oxygen availability, which has a positive effect on Fab 3H6 specific productivities in *P. pastoris*.

Overall, the outcome of this comprehensive study led to new insights into cellular processes that are strongly regulated by oxygen availability. Moreover, the relevance of the data presented in this study has been related to recombinant protein production, pointing out to potential genes or/and metabolic pathways that might be potential targets for strain improvement.

Most notably, while some of adaptations of *P. pastoris *to oxygen availability were consistent with previous transcriptome, proteome and metabolic flux studies reported for *S. cerevisiae*, distinct traits were identified which were, to our knowledge, previously unreported. In particular, the integration of data from three different levels of information for the core metabolism of *P. pastoris *revealed a strong transcriptional induction of glycolysis and the non-oxidative pentose phosphate pathways, and the downregulation of the TCA cycle under hypoxic conditions; such transcriptional changes showed a clear positive correlation with proteomic data and metabolic fluxes, indicating a strong transcriptional regulation of the carbon metabolism in *P. pastoris*. This observation is in clear contrast to *S. cerevisiae*, where no such correlation, at least for glycolysis and the pentose phosphate pathway, has been found [32,80]. Therefore, it seems plausible that increased specific Fab productivities under hypoxia may be -at least partially- the result of increased transcriptional levels of glycolytic genes (and therefore, of genes under the control of glycolytic promoters such as GAP).

Additionally, a wide range of general stress responses and, in particular, stresses related to protein folding and secretion like the UPR, were affected by oxygen scarcity. Since fine-tuning of the translation machinery has been reported to be a successful tool to improve protein secretion - even it was only based on the modification of a single gene - further target genes for strain engineering might be identified from this study. An even more interesting result pointed to a strong interrelation of oxygen availability and the lipid metabolism. The biosynthesis of important membrane components like ergosterol and sphingolipids include a wide range of oxygen dependent reactions so that alterations in the lipid balance under oxygen scarcity are consistent with existing knowledge. Detailed analyses of the impact of such changes on membrane fluidity, or the crucial role of many intercellular membranes in protein translocation to the cell surface might also led to new insights into the potential interrelations of membrane properties and protein secretion processes, making cell membrane properties and biogenesis a novel candidate for rational engineering of the expression system *P. pastoris*.

Nevertheless, systematic extraction of information from the data presented in this study for future rational design of novel strain engineering strategies requires further integration of multi-scale data into a genome-wide metabolic model scaffold, together with the availability of well annotated genomes, thereby linking genome with phenotype.

## Methods

### Yeast strains and chemostat cultivations

The *Pichia pastoris *strain X-33 pGAPZαA Fab3H6, secreting a the light and heavy chain chains of a human monoclonal antibody Fab fragment under the constitutive GAP (TDH3) promoter and the *S. cerevisiae *alpha-mating factor leader, and its control strain (non producing) were cultivated in a glucose-limited chemostat with a working volume of 1 litre at a dilution rate of 0.1 h^-1^, as previously described by Baumann and co-workers [13]. In brief, cells were grown at 25°C, 700 rpm and pH 5.0 under three different oxygen availability conditions. Oxygen concentrations in the inlet gas stream were 21% (normoxia), 11% (oxygen limitation) and 8% (hypoxia, with ethanol and arabitol production). The lower setpoints were created by replacing the air with nitrogen. Different combinations of setpoints were carried out for the three independent biological replica in order to avoid adaptive effects (see [7] for details). Samples were taken for each physiological equilibrium condition after 5 residence times, with the exception of the hypoxic set point, where a sudden wash-out of the culture was observed starting after 3.5 residence times and, therefore, samples were taken just after 3 residence times. Although all measured macroscopic parameters (including biomass, residual substrate and by-product concentrations, CO_2 _production and O_2 _consumption rates) were apparently constant for at least 2 residence times prior to the sudden wash-out, i.e. indicating that cells could sustain a growth rate of 0.1 h^-1 ^during this period, we refer to the hypoxic state as pseudo-steady state.

### Sampling for DNA microarray- and 2D-DIGE analysis from *P. pastoris *chemostat cultures

For DNA microarray analysis, a 9 mL chemostat sample was directly transferred into a pre-chilled Falcon tube containing 5 mL of a freshly prepared, ice cold 5% (v/v) phenol solution in absolute ethanol (Sigma Aldrich). After thorough mixing of the cell suspension, aliquots of 1.5 mL were pelletized by centrifugation at 4°C and immediately stored at -80°C.

For 2D-DIGE analysis, the chemostat sample was divided into six 2 mL aliquots. Cells were collected by centrifugation at 4°C, then the supernatant was stored at -20°C for other analysis, and the pellet was immediately frozen at -80°C.

### Protein analysis by 2D-DIGE

Total protein extraction, DIGE labelling, first and second dimension of the two-dimensional gels as well as data acquisition, data analysis and spot identification were carried out as described by Dragosits and co-workers [7]. In order to prevent dye-specific bias effects in the protein abundance measurements, every individual protein sample (50 μg) was labelled with both Cy3 and Cy5, also known as dye swap correction. The reference pool (mixture of the equal amounts of all individual samples, yielding 50 μg) was labelled with Cy2. Given that two biological replicas of each condition (normoxic, oxygen-limiting and hypoxic) were labelled once by Cy3 and once by Cy5, we run 6 gels per strain and generated 12 measurements for each spot. The ratio of the spot volume of the individual samples (Cy3 or Cy5) and the reference pool (Cy2) was determined to obtain the relative abundance of a protein under each oxygen condition across multiple gels. An average ratio of ≥ 1.5 and a 1-ANOVA *p*-value cut-off of 0.05 were the statistical parameters for the determination of proteins whose abundance was significantly different among two groups (in this case, two oxygen set points). The relative protein abundance profiles of these protein spots were illustrated through PCA and heat map clustering using the R software version 2.6.2 http://www.R-project.org.

### DNA microarrays

The *P. pastoris *DNA microarray used in this study was developed by Graf et al. [71]. RNA extraction, cDNA synthesis and labelling, as well as the microarray hybridizations and data analysis were carried out as reported earlier [71]. All samples were labelled in a dye-swap manner and hybridized against a reference cDNA, which was generated from a pool of cells grown under different culture conditions. Microarray data are available in the ArrayExpress database http://www.ebi.ac.uk/arrayexpress under accession number E-MEXP-2742.

### Validation of microarrays by qRT-PCR

From the analysis of the microarray data, we selected 14 candidate genes with a significant log2 fold change, either between two oxygen setpoints or between two strains, to be validated by qRT-PCR. One reference gene shown to be equally expressed in all samples, β-actin (*ACT1*), was chosen for the relative quantification of expression levels. All primer sequences and primer characteristics (amplicon size, Tm and GC content) are given in Additional file 6.

#### cDNA generation and primer design

For the generation of first strand cDNA, RNA extractions from normoxic and hypoxic chemostat samples from the control and the expressing strain were subjected to a DNAse I (Invitrogen) treatment prior to reverse transcription with SuperScript^®^VILO cDNA Synthesis Kit (Invitrogen). All steps were performed following the manufacturer's protocol, starting from 1 μg RNA. cDNAs were finally filled up to 100 μL (1:5 dilution) with DEPC treated water (Invitrogen). Oligonucleotides (purchased from biomers.net) were designed with Primer Select 7.0.0 (DNASTAR) considering an amplicon size of 100 - 200 bp and a T_m _of approximately 60°C.

#### Primer validation and amplicon purification for standard curve

To guarantee that each primer pair yields a single PCR product of the predicted size, we performed a conventional PCR and confirmed the absence of any primer dimers or unspecific products on a 2% (w/v) agarose gel. To additionally check the specificity of the assay, a melt-curve analysis was performed at the end of each PCR assay. An optimized reaction should have a single peak in the melt-curve, corresponding to the single band on the agarose gel. The specific PCR products were purified (Wizard^®^SV Gel and PCR Clean-Up System, Promega) and quantified on a Nanodrop™3300 (Thermo Scientific). From the concentration and the size of the amplicon, the copy number per μL was determined according to Whelan [81] and decimal dilutions representing 10^7 ^- 10^4 ^copies of target DNA were prepared for the generation of the standard curves.

#### qRT-PCR assay

Quantitative real-time PCR was carried out in 20 μL reactions using semi-skirted iQ 96-well PCR plates and iQ™SYBR^® ^Green supermix (both from Bio-Rad). Samples were measured in triplicates and standards were measured in duplicates on the iCycler Thermal Cycler (Bio-Rad). A non template control was run in every experiment for each of the primer pairs to avoid detection of unspecific priming. The reactions were incubated at 95°C for 5 min to activate the *Taq *polymerase, and then subjected to a three-step cycling protocol including melting (94°C, 15 sec), annealing (58°C, 15 sec) and extension (72°C, 30 sec) for a total of 40 cycles. Each extension was followed by data collection at 72°C and a short incubation step at 78°C (1 sec) for a second plate read closer to the melting point. After a final extension of 5 min at 72°C, we generated a melt-curve profile by data collection during 70 cycles starting at 60°C, with 0.5°C increments/cycle (1-sec intervals).

#### Data analysis

The relative gene expression was calculated for each sample with three measurements giving a maximum standard deviation of 10%. Since the amplification efficiencies of the target and reference genes were not the same in our experiments, we used the Pfaffl method [82] for the relative quantification of our qRT-PCR results.

### Analytical procedures

Cell biomass was monitored by measuring the optical density at 600 nm (OD_600_). For cellular dry weight, a known volume of cultivation broth was filtered using pre-weighted filters; these were washed with two volumes of distilled water and dried to constant weight at 105°C for 24 h. Samples for extracellular metabolite analyses were centrifuged at 6,000 rpm for 2 min in a microcentrifuge to remove the cells and subsequently filtered through 0.45 μm-filters (Millipore type HAWP). Glucose, organic acids, ethanol and arabitol were analyzed by HPLC (Series 1050, Hewlett Packard) with an ionic exchange column (Bio-Rad, Aminex HPX-87H). As mobile phase, 15 mM sulphuric acid was used. The metabolites were detected (Detector HP 1047A, Hewlett Packard) and quantified with the Software EmpowerProfor. The exhaust gas of the bioreactor was cooled in a condenser at 2-4°C (*Frigomix R*, B. Braun Biotech) and dried through a silica gel column. Concentrations of oxygen and carbon dioxide in the exhaust gas of bioreactor cultivations were determined on line with specific sensors (BCP-CO_2 _and BCP-O_2_, BlueSens, Germany).

### Biosynthetically directed fractional (BDF) ^13^C-labelling

*P. pastoris *cells were continuously fed with a minimal medium for five residence times until reaching a metabolic steady state, as indicated by a constant cell density and constant oxygen and carbon dioxide concentrations in the bioreactor exhaust gas. Biosynthetically directed fractional ^13^C labelling (BDF) of cells growing at steady state on a single carbon source has been described elsewhere [20,25,83]. After reaching the steady state, 10% (w/w) of the carbon source in the medium was replaced with uniformly ^13^C-labelled substrate (^13^C-labelled glucose, isotopic enrichment of >98%, from Cortecnet, Voisins le Bretonneux, France). After one residence time, labelled cells were harvested by centrifugation at 4,000 × g for 10 min, resuspended in 20 mM Tris·HCl (pH 7.6) and centrifuged again. Finally, the washed cell pellets were lyophilized (Benchtop 5L Virtis Sentry). An amount of 100 mg of the lyophilized biomass was resuspended in 6 mL of 6 M HCl and subsequently hydrolysed in sealed glass tubes at 110°C for 21 h. The resulting suspensions were filtered using 0.2 μm-filters (Millex-GP, Millipore) and lyophilized. The lyophilized hydrolysates were dissolved in D_2_O for NMR experiments, the pH of the samples being below 1 due to residual HCl.

### NMR spectroscopy and metabolic flux ratio (METAFoR) analysis

2D [^13^C,^1^H]-COSY spectra were acquired for both aliphatic and aromatic resonances as described [84] at 40°C on a Varian Inova spectrometer operating at a ^1^H resonance frequency of 600 MHz. The spectra were processed using the standard Varian spectrometer software VNMR (version 6.1, C). The program FCAL [85] was used for the integration of ^13^C-^13^C scalar fine structures in 2D [^13^C,^1^H]-COSY, for the calculation of relative abundances, *f*-values (see Additional file 4), of intact carbon fragments arising from a single carbon source molecule [84], and for the calculation of the resulting flux ratios through several key pathways in central metabolism, as described by Maaheimo [26] and Jouhten [22].

As described previously [20,25,26,83-87], the calculation of metabolic flux ratios when using fractional ^13^C-labelling of amino acids is based on assuming both a metabolic and an isotopomeric steady state. To establish a cost-effective protocol for a larger number of ^13^C labelling experiments, we fed a chemostat operating in metabolic steady state for the duration of one volume change with the medium containing the ^13^C-labelled substrates [25,83] before harvesting the biomass. Then, the fraction of unlabeled biomass produced prior to the start of the supply with ^13^C-labelled medium can be calculated following simple wash-out kinetics ([86], see also [20] for additional discussion).

### ^13^C-constrained metabolic flux analysis (^13^C-MFA)

Intracellular metabolic fluxes were determined using ^13^C-NMR derived flux ratios as additional experimental constraints to solve the MFA system [88]. The biochemical reaction network model (see Additional files 7 and 8) was based on the stoichiometric model of central carbon metabolism formulated for *S. cerevisiae *[22,26], and *P. pastoris *[20], and adapted conveniently. Briefly, the model included the glycolytic and the pentose phosphate pathways, the TCA cycle and the fermentative pathways, production of glycerol, arabitol, and anabolic fluxes from metabolic intermediates to biosynthesis. The glyoxylate cycle, the PEP carboxykinase and the malic enzyme activity were omitted from the stoichiometric model since the METAFoR data showed that those pathways were either inactive or at basal levels (see [22] for details on the identification of these activities). Separate pools of Pyr, AcCoA and Oaa in the two cellular compartments, cytoplasm and mitochondria, were considered in the metabolic flux ratio analysis. Transports of Pyr and Oaa across the mitochondrial membrane were included in the model but transport of AcCoA, the final step of the cytosolic pyruvate dehydrogenase (PDH) bypass, was omitted. The potential carbon flux through the PDH bypass was lumped into the flux through the PDH reaction, since the ^13^C-labelling protocol used in this study does not allow for an assessment of the split flux ratio between these two pathways (that is, given that flux through malic enzyme is essentially zero in our model, labelled Pyr being metabolized through the PDH bypass does not produce labelling patterns in mitochondrial AcCoA that are distinct from those generated when Pyr is channelled through the PDH reaction. Nevertheless, flux through the PDH bypass cannot be totally excluded, as discussed in the Results section.

The complete model for the calculation of intracellular fluxes, comprised 33 (normoxic condition) and 34 (oxygen-limiting and hypoxic conditions) metabolic reactions. The measured uptake and excretion rates and the rates of metabolic precursor depletion to biosynthesis, as determined from the composition of *P. pastoris *biomass previously reported for each oxygenation condition [18], were combined with a set of linearly independent equations obtained from METAFoR analysis to render the complete linear system solvable.

The determined metabolic flux ratios were used as additional constraints for solving the metabolic network following a ^13^C constrained flux balancing approach similarly to a previous approach [88]. Using the constraints from the METAFoR analysis, it was not necessary to include redox cofactor mass balances. Cofactor mass balances are sources of errors since the correct balancing requires detailed knowledge of the relative activities of different isoenzymes and the enzyme cofactor specificities on a cell wide scale. The flux ratios considered in the present approach were the following (equations 1 to 4, the reaction numbers are defined in Additional file 8):

the fraction of Oaa_mit _originating from Oaa_cyt_, that is, Oaa_cyt _transport into the mitochondria (only applicable to oxygen-limiting and hypoxic conditions):

(1) $$a=\frac{x_{23}}{x_{23}+x_{16}}$$

Under normoxic conditions, reaction *x*_*24 *_(flux of Oaa_cyt _into the mitochondria) was calculated as a *net *Oaa transport flux across the mitochondrial membrane (that is, Oaa_cyt _import - Oaa_mit _export), annotated as *x*_24_*. Also, the labelling patterns of Pep instead of Pyr_cyt _were considered (see Results section) and, therefore, the corresponding anaplerotic flux ratio was defined as:

(2) $$b=\frac{x_{23}*}{x_{23}*+x_{16}}$$

the fraction of Oaa_cyt _originating from Pyr_cyt_, that is, the anaplerotic flux ratio (only applicable to oxygen-limiting and hypoxic conditions):

(3) $$c=\frac{x_{17}}{x_{17}+x_{24}}$$

the fraction of Pep from PPP assuming a maximal contribution of PPP:

(4) $$d\geq \frac{x_{9}+2\left(x_{11}\right)+3\left(x_{10}\right)}{2\left(x_{3}\right)+x_{9}+x_{10}}$$

The following linear constraint equations (equations 5 to 8) were derived from the flux ratio equations:

(5) $$x_{23}\left(1-a\right)+x_{16}\left(-a\right)=R_{a}$$

(6) $$x_{23}*\left(1-b\right)+x_{16}\left(-b\right)=R_{b}$$

(7) $$x_{17}\left(1-c\right)+x_{24}\left(-c\right)=R_{c}$$

(8) $$x_{9}\left(1-d\right)+x_{11}\left(2\right)+x_{10}\left(3-d\right)+x_{3}\left(-2\right)\leq R_{d}$$

Equations 5 to 7 were added to the stoichiometric model as a submatrix F, obtaining the complete metabolic model to solve the metabolite mass balances:

(9) $$\left[\begin{array}{c} S\\ F \end{array}\right]\cdot x=\left[\begin{array}{c} c\\ 0 \end{array}\right]\equiv N\cdot x=b$$

where S represents the stoichiometric matrix (including input/output reactions), c is a column vector with either 0 for internal reactions or the corresponding value for each one of the input/output rates and × is the vector of fluxes. Solution of the resulting linear system was obtained using the MATLAB function *lsqlin *using equation 8 as an additional constrain. Irreversibility was assumed for several intracellular fluxes and for the depletion of precursors to biosynthetic reactions, that is, only positive values were allowed for these fluxes (see Additional file 8).

Confidence intervals for the optimized fluxes were calculated up on the determination of their standard deviation using the Fisher Information Matrix approach (FIM) [89] as:

(10) $$\sigma_{j}={\sqrt{\left(FIM^{-1}\right)}}_{jj}$$

Calculation of FIM was performed as:

(11) $$FIM=\sum W^{T}C^{-1}W$$

where C is the variance-covariance matrix of the measurements (assumed independent) and W is a parameter sensitivity matrix where each element of w_ij _corresponds to:

(12) $$w_{ij}=\frac{\partial x_{i}}{\partial p_{j}}$$

which describes an infinitesimal change of the variable x_i _(e.g. a measurement) due to an infinitesimal change in parameter p_j _(a flux).

Confidence intervals for the estimated fluxes ${\hat{p}}_{j}$ of *p*_*j *_can be derived [90] from:

(13) $${\hat{p}}_{j}-\sigma_{p_{j}}.t_{\alpha /2}^{v}<p_{j}<{\hat{p}}_{j}+\sigma_{p_{j}}.t_{\alpha /2}^{v}$$

where $t_{\alpha /2}^{v}$ corresponds to the Student's t distribution, with v degrees of freedom and α corresponds to the (1-α) confidence interval chosen. All calculations were performed on a PC compatible computer running Matlab ^® ^7.4 (v2007b) for Windows.

## Authors' contributions

KB performed bioreactor cultivations, proteomics and microarray experiments, quantitative real-time PCR, data analysis and interpretation of the results, and drafted the manuscript. MC carried out the ^13^C-labeling experiments and macroscopic data processing, performed the metabolic flux calculations and participated in interpretation of results. MD assisted in the design and performance of proteome and microarray experiments. ABG participated in the design and bioinformatics analysis of the microarrays. JS carried out the protein identification by liquid chromatography-tandem mass spectrometry. PJ and HM performed the 2D-NMR and METAFoR analyses and participated in the subsequent interpretation of results. BG helped with the conceptual design of the study and with the interpretation of omics data. JA designed the ^13^C-constrained MFA approach, and participated in analysis and interpretation of MFA results. DM participated in the overall conceptual and experimental design of this study and interpretation of results. PF participated in the conceptual and experimental design, interpretation of results and in drafting the manuscript. All authors read and approved the final manuscript.

## Supplementary Material

Additional file 1 **Design 2D DIGE Gels**. An example of the experimental design for the acquisition of statistical data on differences between samples taken from normoxic (21%), oxygen-limiting (11%) and hypoxic (8%) setpoints. Replica of 2 independent experiments (F1 or F2; F = fermentation) were labelled with either Cy5 or Cy3 (GE Healthcare). A pool of all samples served as reference and was labelled with Cy2 (= pooled standard). Click here for file

Additional file 2 **2D DIGE Gels**. Representative gel image from a 2D gel electrophoresis experiment with proteins obtained from the Fab-expressing strain. We identified 45 out of 81 proteins with a different expression pattern when comparing high and low oxygen experiments. Green spots show proteins downregulated and pink ones show those upregulated under hypoxia. Click here for file

Additional file 3 **Identified protein spots**. List of the 45 identified proteins with different abundances comparing hypoxic (8) and normoxic (21) conditions in the *P. pastoris *expressing and control strain. Proteins were identified by MALDI-TOF MS and grouped into 6 different biological processes. The protein name, short name and accession number, theoretical Mw and pI are reported together with the percentage of peptide coverage and number of identified peptides. Average ratios and 1-ANOVA (DeCyder) are given and only not indicated where no spot could be matched. p.i. = previously identified on other 2D gels Click here for file

Additional file 4 **Relative abundances of intact carbon fragments in proteinogenic amino acids**. Relative abundances of intact C2 and C3 fragments (*f*-values) in proteinogenic amino acids describing the conservation of carbon chain fragments in *P. pastoris *Fab-producing and control strains growing in glucose-limited chemostats at a D = 0.1 h^-1 ^in different oxygenation conditions. Click here for file

Additional file 5 **Metabolic fluxes** Metabolic fluxes in the central carbon metabolism of *P. pastoris *Fab-producing and control strain in glucose-limited chemostats at a D = 0.1 h^-1 ^in different oxygenation conditions. The standard deviations of each net flux are given. Click here for file

Additional file 6 **qRT-PCR primer sequences used in this study**. Table showing the primer sequences of the genes analyzed by qRT-PCR and characteristics of the corresponding amplicons. Calculated copy numbers and the dilution factors in order to obtain 10^9 ^copies μl^-1 ^are indicated (see main text for explanation). Click here for file

Additional file 7 **Metabolic network model of the central carbon metabolism of *P. pastoris***. Bioreaction network model of the central carbon metabolism of *P. pastoris *used in the ^13^C-metabolic flux analysis for the determination of net fluxes under the different oxygenation conditions. Fluxes are represented as net fluxes and the directions of the arrows indicate the directions of the positive net fluxes. The metabolites consumed or produced by extracellular fluxes (shown as dashed arrows) are denoted with (E). Click here for file

Additional file 8 **Stoichiometric model of the central carbon metabolism of *P. pastoris***. Reactions in the stoichiometric model of the central carbon metabolism of *P. pastoris *applied in the ^13^C-MFA determination of the metabolic fluxes under different oxygenation conditions; it also includes anabolic reactions from metabolic intermediates to biosynthesis, transport reactions across the mitochondrial membrane and uptake and excretion reactions. Note that O_2_, CO_2_, energy and redox cofactor mass balances were not included in the mass balance constraints for ^13^C-MFA. Click here for file
