# Supplementary material for: A multi-level study of recombinant Pichia pastoris in different oxygen conditions
Source: BMC Syst Biol. 2010 Oct 22;4:141. doi: 10.1186/1752-0509-4-141 (PMC2987880; doi:10.1186/1752-0509-4-141)
Supplement: Additional file 1 — Design 2D DIGE Gels. An example of the experimental design for the acquisition of statistical data on differences between samples taken from normoxic (21%), oxygen-limiting (11%) and hypoxic (8%) setpoints. Replica of 2 independent experiments (F1 or F2; F = fermentation) were labelled with either Cy5 or Cy3 (GE Healthcare). A pool of all samples served as reference and was labelled with Cy2 (= pooled standard). [file 1752-0509-4-141-S1.DOC]

| **Gel** | **Cy2** | **Cy3** | **Cy5** |
| --- | --- | --- | --- |
| **1** | pooled standard | 21% F1 | 11% F1 |
| **2** | pooled standard | 21% F2 | 08% F2 |
| **3** | pooled standard | 08% F1 | 11% F2 |
| **4** | pooled standard | 11% F2 | 21% F2 |
| **5** | pooled standard | 11% F1 | 08% F1 |
| **6** | pooled standard | 08% F2 | 21% F1 |
