# Supplementary figures and images for: A multi-level study of recombinant Pichia pastoris in different oxygen conditions
Source: BMC Syst Biol. 2010 Oct 22;4:141. doi: 10.1186/1752-0509-4-141 (PMC2987880; doi:10.1186/1752-0509-4-141)

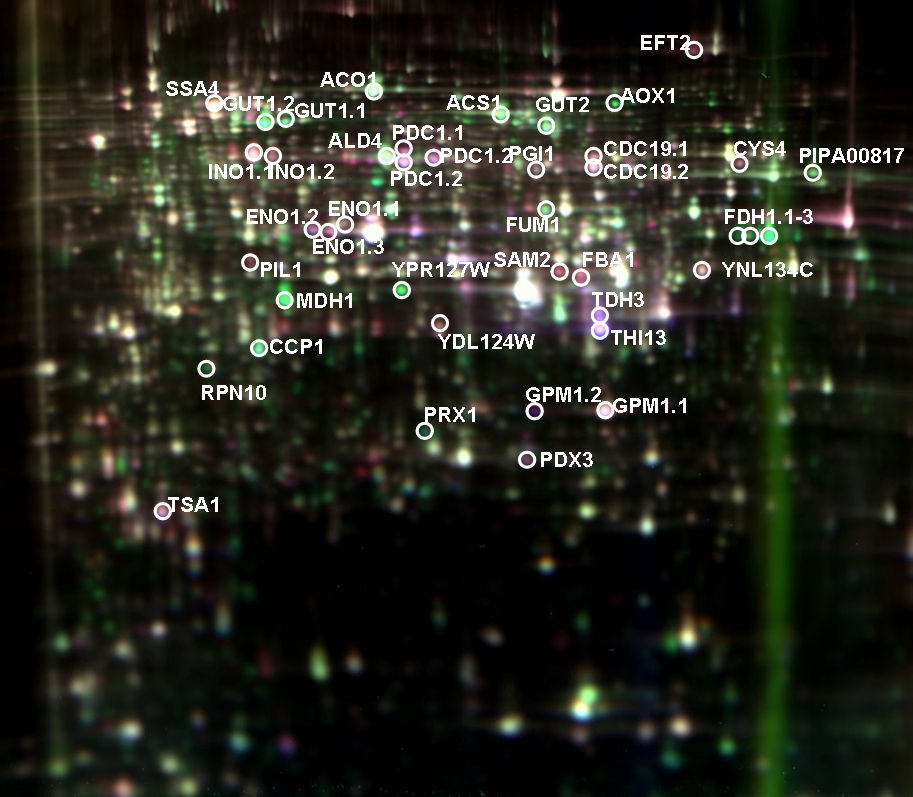

Supplement: Additional file 2 — 2D DIGE Gels. Representative gel image from a 2D gel electrophoresis experiment with proteins obtained from the Fab-expressing strain. We identified 45 out of 81 proteins with a different expression pattern when comparing high and low oxygen experiments. Green spots show proteins downregulated and pink ones show those upregulated under hypoxia. [file 1752-0509-4-141-S2.PNG]
