# Supplementary material for: A multi-level study of recombinant Pichia pastoris in different oxygen conditions
Source: BMC Syst Biol. 2010 Oct 22;4:141. doi: 10.1186/1752-0509-4-141 (PMC2987880; doi:10.1186/1752-0509-4-141)
Supplement: Additional file 3 — Identified protein spots. List of the 45 identified proteins with different abundances comparing hypoxic (8) and normoxic (21) conditions in the P. pastoris expressing and control strain. Proteins were identified by MALDI-TOF MS and grouped into 6 different biological processes. The protein name, short name and accession number, theoretical Mw and pI are reported together with the percentage of peptide coverage and number of identified peptides. Average ratios and 1-ANOVA (DeCyder) are given and only not indicated where no spot could be matched. p.i. = previously identified on other 2D gels [file 1752-0509-4-141-S3.DOC]

| **Protein Data** | | | | | | | | | | | | | |
| --- | --- | --- | --- | --- | --- | --- | --- | --- | --- | --- | --- | --- | --- |
|  | Short name | Protein name | Protein ID | MS Data | | | | Expressing 8.21 | | | Control 8.21 | | |
|  |  |  |  | Mass kDa | pI | coverage | Peptide matches | Spot Nº | Av.  ratio | 1-ANOVA | Spot Nº | Av.  ratio | 1-ANOVA |
| GLYCOLYSIS | CDC19.1 | Pyruvate kinase | PIPA00751 | 49.56 | 6.62 | 34.5 | 15 | 712 | 1.91 | 8.00E-03 | 604 | 3.14 | 9.80E-03 |
|  | CDC19.2 |  | PIPA00751 | 49.56 | 6.62 | 32.5 | 9 | 769 | 2.19 | 9.20E-04 | 854 | 2.98 | 2.80E-05 |
|  | PGI1 | Phosphoglucose isomerase | PIPA03258 | 61.94 | 6.16 | 4.8 | 2 | 788 | 1.88 | 7.10E-03 | 845 | 2.34 | 1.20E-03 |
|  | ENO1.1 | Enolase | PIPA00938 | 46.49 | 5.55 | 26.8 | 8 | 1054 | 1.69 | 2.60E-02 | 837 | 2.86 | 2.60E-04 |
|  | ENO1.2 |  | PIPA00938 | 46.49 | 5.55 | 45.2 | 15 | 1116 | 2.45 | 6.60E-04 | 1122 | 1.57 | 1.80E-04 |
|  | ENO1.3 |  | PIPA00938 | 46.49 | 5.55 | 54.4 | 21 | 1117 | 2.4 | 1.50E-02 | 1116 | 2.09 | 1.90E-05 |
|  | TDH3 | Glyceraldehyde 3-phosphate dehydrogenase | PIPA02510 | 30.92 | 5.81 | 15.9 | 3 | 1477 | 1.51 | 2.40E-02 | 1127 | 2.06 | 8.20E-04 |
|  | FBA1 | Fructose 1,6-bisphosphate aldolase | PIPA00092 | 39.7 | 6.38 | 26.3 | 5 | 1354 | 1.72 | 1.20E-01 | 1039 | 3.72 | 8.60E-03 |
|  | GPM1.1 | Phosphoglycerate mutase | PIPA02723 | 22.42 | 5.15 | 44.0 | 8 | 1829 | 1.96 | 2.10E-02 | 1610 | 2.12 | 5.20E-04 |
|  | GPM1.2 |  | PIPA02723 | 22.42 | 5.15 | 23 | 4 | 1830 | 3.38 | 8.00E-03 | 1605 | 1.98 | 3.30E-03 |
| TCA | ACO1 | Aconitase | PIPA00361 | 84.5 | 5.39 | 36.3 | 23 | 282 | -1.61 | 3.50E-03 | 478 | -1.87 | 1.10E-03 |
|  | FUM1 | Fumarase | PIPA02844 | 52.6 | 6.79 | 32.4 | 12 | 1028 | -1.33 | 4.10E-04 | 1040 | -1.58 | 3.20E-04 |
|  | MDH1 | Mitochondrial malate dehydrogenase | PIPA02244 | 32.67 | 4.96 | 43.2 | 15 | 1466 | -4.02 | 1.60E-03 | 1382 | -6.81 | 1.40E-06 |
| VITAMIN METAB. PROCESS | ALD4 | Mitochondrial aldehyde dehydrogenase | PIPA00390 | 42.15 | 8.45 | 39.6 | 11 | 711 | -2.15 | 1.90E-04 | 797 | -2.14 | 7.60E-06 |
|  | FDH1.1 | NAD(+)-dependent formate dehydrogenase | PIPA03425 | 40.29 | 7.05 | 10.4 | 2 | 1130 | -2.09 | 1.00E-03 | - | - | - |
|  | FDH1.2 |  | PIPA03425 | 40.29 | 7.05 | 15.9 | 3 | 1166 | -1.87 | 6.10E-04 | - | - | - |
|  | FDH1.3 |  | PIPA03425 | 40.29 | 7.05 | 15.9 | 3 | 1170 | -2.32 | 2.00E-03 | - | - | - |
|  | FDH1.4 |  | PIPA03425 | 40.29 | 7.05 | 31.8 | 8 | 1175 | -3.91 | 1.70E-03 | 1148 | -8.36 | 4.00E-05 |
|  | GUT2.1 | Mitochondrial glycerol-3-phosphate dehydrogenase | PIPA02567 | 31.72 | 9.11 | 24.4 | 5 | 514 | -1.45 | 1.30E-02 | 593 | -2.04 | 3.10E-04 |
|  | GUT2.2 |  | PIPA02567 | 44.80 | 6.14 | 38.5 | 13 | 514 | -1.45 | 1.30E-02 | 593 | -2.04 | 3.10E-04 |
|  | GUT2.3 |  | PIPA02567 | 37.53 | 5.93 | 41.8 | 12 | 514 | -1.45 | 1.30E-02 | 593 | -2.04 | 3.10E-04 |
| STRESS RESPONSE | PRX1 | Mitochondrial thioredoxin peroxidase | PIPA01363 | 25.12 | 6.02 | 43.4 | 8 | 1887 | -1.96 | 1.40E-02 | 1690 | -2.16 | 2.10E-03 |
|  | SSA4 | Heat shock protein SSA4 | PIPA03073 | 70.72 | 5.12 | 19.6 | 8 | 365 | 1.36 | 4.90E-02 | 403 | 3.06 | 1.90E-02 |
|  | TSA1 | Thioredoxin peroxidase | PIPA04168 | 36.42 | 5.26 | 19.6 | 4 | 2077 | 1.84 | 1.50E-02 | 1832 | 1.73 | 2.90E-04 |
|  | PIL1 | Hypothetical protein | PIPA00237 | 35.22 | 5.07 | 9.3 | 2 | 1377 | 1.35 | 1.90E-01 | 1005 | 2.2 | 9.30E-03 |
|  | CCP1 | Mitochondrial cytochrome-c peroxidase | PIPA04202 | 41.9 | 7.01 | p.i. | p.i. | 1645 | -1.42 | 1.30E-02 | 1503 | -2.11 | 1.40E-03 |

| **Protein Data** | | | | | | | | | | | | | |
| --- | --- | --- | --- | --- | --- | --- | --- | --- | --- | --- | --- | --- | --- |
|  | Short name | Protein name | Protein ID | MS Data | | | | Expressing 8.21 | | | Control 8.21 | | |
|  |  |  |  | Mass kDa | pI | coverage | Peptide matches | Spot Nº | Av.  ratio | 1-ANOVA | Spot Nº | Av.  ratio | 1-ANOVA |
| AMINO ACID METAB. | CYS4 | Cystathionine beta-synthase | PIPA04214 | 54.89 | 7.16 | 2.4 | 1 | 752 | 1.63 | 2.30E-02 | 844 | 1.91 | 1.10E-03 |
|  | PDC1.1 | Pyruvate decarboxylase | PIPA01726 | 61.32 | 5.88 | 36.4 | 15 | 665 | 2.16 | 3.90E-03 | - | - | - |
|  | PDC1.2 |  | PIPA01726 | 61.32 | 5.88 | 17.1 | 6 | 740 | 1.99 | 6.10E-03 | 802 | 1.25 | 1.40E-02 |
|  | PDC1.3 |  | PIPA01726 | 61.32 | 5.88 | 54.5 | 25 | 746 | 3.07 | 4.40E-04 | 629 | 6.31 | 2.60E-03 |
|  | THI3 | Pyrimidine precursor biosynthesis enzyme thi3 | PIPA00420 | 38.22 | 6.63 | 18.2 | 6 | 1588 | 3.04 | 3.40E-02 | 1188 | 3.11 | 1.50E-01 |
|  | SAM2 | S-adenosylmethionine synthetase | PIPA00230 | 42.3 | 6.43 | p.i. | p.i. | 1333 | 1.2 | 4.30E-02 | 1249 | 2.88 | 1.60E-03 |
| OTHERS | RPN10 | 19S/PA700 proteasome regulatory particle subunit Rpn10p/S5a | PIPA03368 | 29.66 | 5.15 | 16.1 | 2 | 1703 | -1.74 | 3.90E-03 | 1583 | -1.89 | 2.00E-03 |
|  | EFT2 | Elongation factor 2 (EF-2) | PIPA00576 | 93.40 | 6.63 | 9.1 | 6 | 111 | 1.28 | 2.30E-02 | 165 | 2.11 | 2.00E-02 |
|  | GUT1.1 | Glycerol kinase | PIPA01632 | 68.15 | 5.33 | 2.4 | 1 | 477 | -2.41 | 2.20E-03 | 644 | -3.88 | 2.60E-04 |
|  | GUT1.2 |  | PIPA01632 | 68.15 | 5.33 | 42.0 | 19 | 483 | -2.72 | 2.10E-03 | 650 | -5.50 | 1.10E-06 |
|  | INO1.1 | Myo-inositol-1-phosphate synthase | PIPA04248 | 58.40 | 5.26 | 34.7 | 13 | 679 | 1.39 | 1.80E-03 | 791 | 1.79 | 8.90E-03 |
|  | INO1.2 |  | PIPA04248 | 58.40 | 5.26 | 2.1 | 1 | 709 | 2.07 | 1.80E-03 | 784 | 2.27 | 2.60E-04 |
|  | YNL134C | Zinc binding oxidoreductase | PIPA00176 | 37.79 | 6.73 | 24.6 | 7 | 1323 | 1.87 | 1.20E-01 | 1065 | 2.47 | 8.10E-03 |
|  | YPR127W | Pyridoxal reductase? | PIPA01079 | 31.81 | 5.86 | 3.5 | 1 | 1431 | -2.7 | 2.10E-06 | 1308 | -3.2 | 7.50E-04 |
|  | - | acyl-CoA thioester hydrolase | PIPA00817 | 57.74 | 8.65 | 27.1 | 9 | 808 | -2.22 | 8.00E-04 | 886 | -2.64 | 2.60E-04 |
|  | AOX1 | Alcohol oxidase | PIPA01780 | 73.8 | 6.41 | p.i. | p.i. | 348 | -3.58 | 3.60E-03 | 534 | -7.56 | 1.60E-03 |
|  | PDX3 | Pyridoxine (pyridoxamine) phosphate oxidase | PIPA04880 | 26.07 | 6.56 | 18.7 | 3 | 1957 | 2.01 | 2.50E-04 | 1720 | 1.32 | 7.40E-02 |
|  | YDL124W | NADPH-dependent alpha-keto amide reductase | PIPA01263 | 33.69 | 5.57 | 12.4 | 3 | 1566 | 1.63 | 1.60E-02 | 1208 | 2.28 | 1.10E-02 |
|  | ACS1 | Acetyl-coA synthetase isoform | PIPA00753 | 73.8 | 6.02 | p.i. | p.i. | 432 | -2.71 | 4.90E-04 | 602 | -2.01 | 4.10E-04 |
