# Supplementary material for: A multi-level study of recombinant Pichia pastoris in different oxygen conditions
Source: BMC Syst Biol. 2010 Oct 22;4:141. doi: 10.1186/1752-0509-4-141 (PMC2987880; doi:10.1186/1752-0509-4-141)
Supplement: Additional file 7 — Metabolic network model of the central carbon metabolism of P. pastoris. Bioreaction network model of the central carbon metabolism of P. pastoris used in the 13C-metabolic flux analysis for the determination of net fluxes under the different oxygenation conditions. Fluxes are represented as net fluxes and the directions of the arrows indicate the directions of the positive net fluxes. The metabolites consumed or produced by extracellular fluxes (shown as dashed arrows) are denoted with (E). [file 1752-0509-4-141-S7.PPT]

## Slide 1
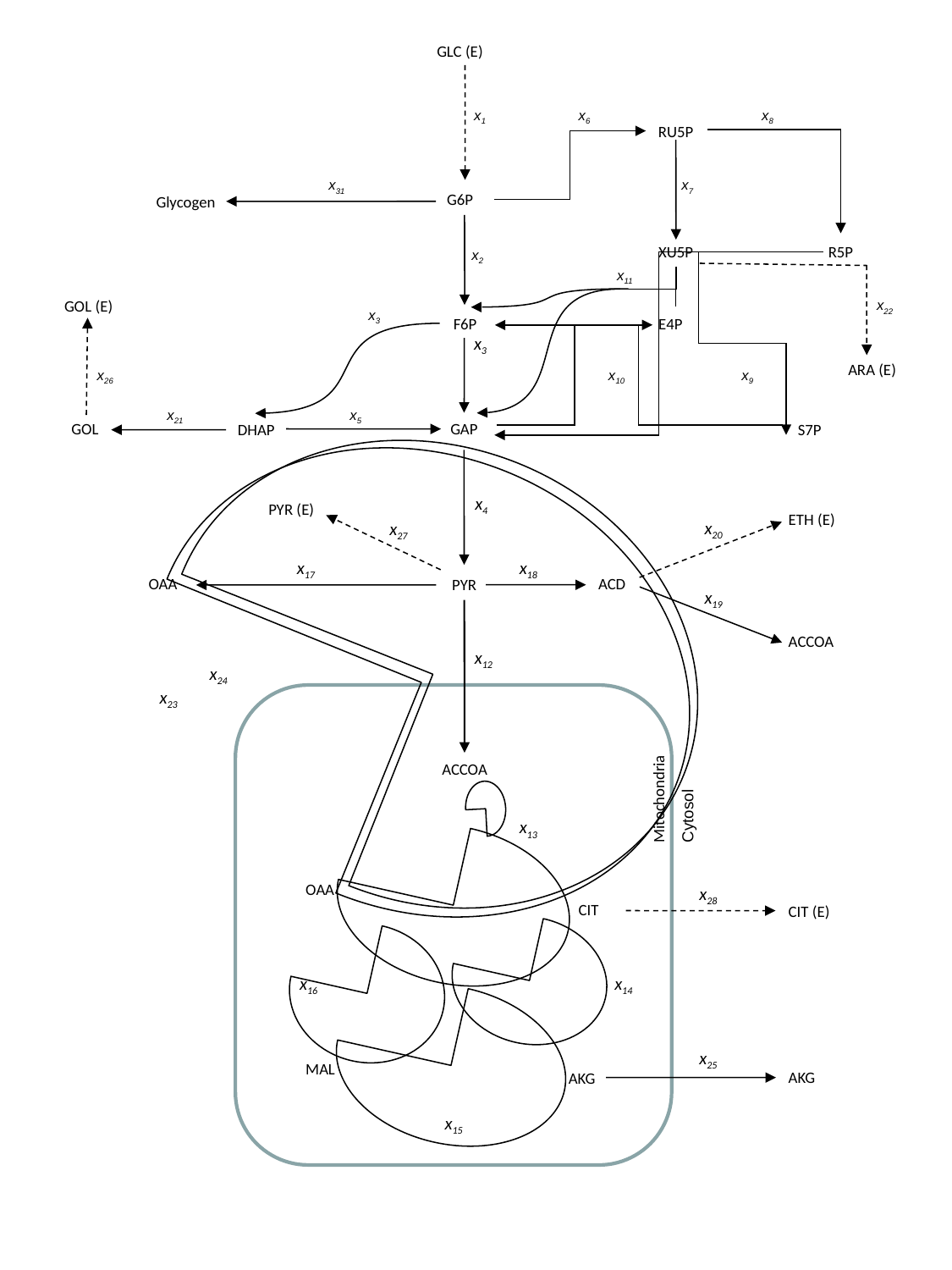

GLC (E)
x1
x6
x8
RU5P
x31
x7
G6P
Glycogen
XU5P
R5P
x2
x11
GOL (E)
x22
x3
F6P
E4P
x3
ARA (E)
x26
x10
x9
x21
x5
GOL
GAP
DHAP
S7P
x4
PYR (E)
ETH (E)
x27
x20
x17
x18
OAA
ACD
PYR
x19
ACCOA
x12
x24
x23
ACCOA
Mitochondria
Cytosol
x13
OAA
x28
CIT
CIT (E)
x16
x14
x25
MAL
AKG
AKG
x15
