# Supplementary material for: A multi-level study of recombinant Pichia pastoris in different oxygen conditions
Source: BMC Syst Biol. 2010 Oct 22;4:141. doi: 10.1186/1752-0509-4-141 (PMC2987880; doi:10.1186/1752-0509-4-141)
Supplement: Additional file 8 — Stoichiometric model of the central carbon metabolism of P. pastoris. Reactions in the stoichiometric model of the central carbon metabolism of P. pastoris applied in the 13C-MFA determination of the metabolic fluxes under different oxygenation conditions; it also includes anabolic reactions from metabolic intermediates to biosynthesis, transport reactions across the mitochondrial membrane and uptake and excretion reactions. Note that O2, CO2, energy and redox cofactor mass balances were not included in the mass balance constraints for 13C-MFA. [file 1752-0509-4-141-S8.DOC]

x_i_ Reaction

Glycolysis

x_1_ Glucose_ext_ + ATP => G6P + ADP

x_2_ G6P <=> F6P

x_3_ F6P + ATP => GAP + DHAP + ADP

x_4_ GAP + 2ADP + NAD^+^ => Pyr + 2ATP + NADH

x_5_ DHAP <=> GAP

Pentose Phosphate Pathway

x_6_ G6P + 2 NADP^+^ => RU5P + 2 NADPH + CO_2_

x_7_ RU5P <=> XU5P

x_8_ RU5P <=> R5P

x_9_ R5P + XU5P< => S7P + GAP

x_10_ E4P + XU5P <=> F6P + GAP

x_11_ S7P + GAP <=> F6P + E4P

Pyruvate Dehydrogenase

x_12_ CoA + NAD^+^ + Pyr => AcCoA_mit_ + NADH + CO_2_

TCA cycle

x_13_ AcCoA_mit_ + Oaa_mit_ => CoA + Cit_mit_

x_14_ Cit_mit_ + NAD^+^ => NADH + CO_2_ + Akg_mit_

x_15_ NAD^+^ + Akg_mit_ + ADP + FAD => Mal + NADH+ FADH_2_ + ATP + CO_2_

x_16_ Mal + NAD^+^ => OAA_mit_ + NADH

Anaplerotic Pathways

x_17_ ATP + CO_2_ + Pyr => Oaa_cyt_ + ADP

Fermentative Pathways

x_18_ Pyr => AcO + CO_2_

x_19_ AcO + NADP^+^ + CoA + ATP => NADPH + AcCoA_cyt_ + Pyrophosphate + AMP

x_20_ AcO + NADH => Eth_ext_ + NAD^+^

x_21_ DHAP + NADH + ATP => Glycerol_cyt_ + Orthophosphate + ADP + NAD^+^

x_22_ XU5P +2 NADH + NADP^+^ => ARA_ext_ + 2 NAD^+^ + NADPH

Cytosol-mitochondria transport reactions

x_23_ Oaa_cyt_ => Oaa_mit_ (in oxygen-limiting and hypoxic conditions)

x_23_* Oaa_cyt_ => Oaa_mit_ (in normoxic condition)

x_24_ Oaa_mit_ => Oaa_cyt_ (in oxygen-limiting and hypoxic conditions)

x_25_ Akg_mit_ => Akg_cyt_

Metabolite excretion reactions

x_26_ Glycerol_cyt_ => Glycerol_ext_

x_27_ Pyr => Pyr_ext_

x_28_ Cit => Cit_ext_

Biomass synthesis reactions for each oxygenation condition

1. Protein (Composition derived from the amino acid composition of each oxygenation condition [18]).

x_29.1_ (21 % O_2_ condition): 0.136 Pyr + 0.006 R5P + 0.013 E4P + 0.031 Oaa_cyt_ + 0.014 AcCoA_mit_ + 0.009 Oaa_mit_ + 0.075 Akg_cyt_ + 0.013 AcCoA_cyt_ + 0.300 NAD^+^ + 1.710 NADPH => 1 C-mol Protein + 0.002 GAP + 0.058 CO_2_ + 0.300 NADH + 1.710 NADP^+^

x_29.2_ (11 % O_2_ condition): 0.141 Pyr + 0.006 R5P + 0.013 E4P + 0.033 Oaa_cyt_ + 0.015 AcCoA_mit_ + 0.009 Oaa_mit_ + 0.070 Akg_cyt_ + 0.014 AcCoA_cyt_ + 0.303 NAD^+^ + 1.704 NADPH => 1 C-mol Protein + 0.002 GAP + 0.066 CO_2_ + 0.303 NADH + 1.704 NADP^+^

x_29.3_ (8 % O_2_ condition): 0.144 Pyr + 0.006 R5P + 0.014 E4P + 0.034 Oaa_cyt_ + 0.016 AcCoA_mit_ + 0.010 Oaa_mit_ + 0.068 Akg_cyt_ + 0.014 AcCoA_cyt_ + 0.239 NAD^+^ + 1.697 NADPH => 1 C-mol Protein + 0.002 GAP + 0.069 CO_2_ + 0.239 NADH + 1.697 NADP^+^

1. Carbohydrate (Composition derived from [91])

x_30_ 0.113 G6P + 0.053 F6P => 1 C-mol Carbohydrate

1. Glycogen

x_31_ 0.166 G6P => 1 C-mol Glycogen

1. Lipid (Composition derived from the mean lipid composition of all conditions [18]).

x_32_ 0.002 G6P + 0.005 Pyr + 0.006 CO_2_ + 0.061 AcCoA_mit_ + 0.386 AcCoA_cyt_ + 0.065 O_2_ + 0.022 Glycerol_cyt_ + 0.06 NADH + 0.77 NADPH => 1 C-mol Lipid + 0.06 NAD^+^ + 0.77 NADP^+^

1. RNA (Composition derived from the RNA composition proposed by [92])

x_33_ 0.060 Pyr + 0.060 CO_2_ + 0.111 R5P + 0.051 Oaa_cyt_ + 0.181 NAD^+^ + 0.079 NADPH => 1 C-mol RNA + 0.181 NADH + 0.079 NADP^+^

1. DNA (Composition derived from the DNA composition published in [31])

x_34_ 0.054 Pyr + 0.085 CO_2_ + 0.108 R5P + 0.054 Oaa_cyt_ + 0.161 NAD^+^ + 0.225 NADPH => 1 C-mol DNA + 0.161 NADH + 0.225 NADP^+^
