# Supplementary material for: A multi-level study of recombinant Pichia pastoris in different oxygen conditions
Source: BMC Syst Biol. 2010 Oct 22;4:141. doi: 10.1186/1752-0509-4-141 (PMC2987880; doi:10.1186/1752-0509-4-141)
Supplement: Supplementary file 8 [file 1752-0509-4-141-S6.DOC]

	oligo sequence		oligo name	T_m	amplicon bp	GC content	amplicon conc. [ng/µl]	copies / µl	dilution to 10^9 copies / µl
5'	CTTGCCAGCTCTTAGTGAAA	3'	fwd CDC19	58.35	122	40%	92.44	6.90E+11	689.51
5'	GAACATGGCGAATCTCCTT	3'	rev CDC19	58.01					
5'	ATCTGCACCACCACTACTTCATTG	3'	fwd ERG25	62.86	131	48%	95.96	6.67E+11	666.59
5'	TCTTTCGGCCTTTAGCTTCATACG	3'	rev ERG25	62.86					
5'	GAGGCCAGTCGACATATCATTCAG	3'	fwd FLR1	64.57	163	38%	96.18	5.37E+11	536.96
5'	CACGCAGAGCATATTAAGACACCA	3'	rev FLR1	64.55					
5'	TGAAGCCAACCCAGACAGA	3'	fwd FUM1	60.16	164	47%	114.62	6.36E+11	636.00
5'	AAAGTACGCCCAGTTCCAGAG	3'	rev FUM1	62.57					
5'	TGTCGTTGAGCCATCTTTCGTC	3'	fwd MDH1	62.67	155	48%	110.74	6.50E+11	650.15
5'	TGGCAGTTGTGATCATCTCCTCTT	3'	rev MDH1	62.86					
5'	CAAGGACCCCAAAGAAAAGTGTGC	3'	fwd NCE103	64.57	174	48%	108.04	5.65E+11	565.04
5'	CATGGGATCTCAACCTCGCTCAA	3'	rev NCE103	64.55					
5'	ACTCATCCAGCCCCCATCATCAT	3'	fwd AQR1	64.55	159	47%	95.61	5.47E+11	547.20
5'	TAGGGCGCTCATTAGGCATCTTGT	3'	rev AQR1	64.57					
5'	GATATTGCATTTGACGGGGCTGAT	3'	fwd RKI1	62.86	125	46%	79.25	5.77E+11	576.94
5'	TCTGCGACGATGAATAGTTGTTT	3'	rev RKI1	59.2					
5'	TGCCTGAACAATACCGTTCC	3'	fwd PFK3	62.45	174	48%	85.72	4.48E+11	448.31
5'	AGTCTGTTTCACGGTCATCC	3'	rev PFK3	61.54					
5'	AGATGAGTTGCCTTTGCTGGATT	3'	fwd YCT1	60.99	146	39%	122.56	7.64E+11	763.90
5'	AGTTTCATTGCCTTCGGTTTGTTA	3'	rev YCT1	59.44					
5'	TTGGCCAAAAATATCGGAGTC	3'	fwd YDR124W	58.66	193	46%	108.11	5.10E+11	509.74
5'	ACAATGGAGCCAAAGGAGAGTAA	3'	rev YDR124W	60.99					
5'	TGTCCGGTGGTACTACTATGTTCC	3'	fwd ACT1	64.57	199	47%	110.65	5.06E+11	505.99
5'	GATTCGTCGTACTCTTGCTTTGA	3'	rev ACT1	60.99					
5'	GTAAGCCTTGTGGGTAGAGTC	3'	fwd TDH3	62.57	165	47%	122.53	6.76E+11	675.77
5'	ATGGCTATCACTGTCGGTATT	3'	rev TDH3	58.66					
5'	CTATTACTGTGCAAGAACGTC	3'	fwd Fab Hc	60.21	132	59%	61.76	4.26E+11	425.77
5'	GAGGAGGGTGCCAGGGG	3'	rev Fab Hc	59.69					
5'	CTCCAATCGGGTAACTC	3'	fwd Fab Lc	60.35	131	55%	81.24	5.64E+11	564.34
5'	GTGACTTCGCAGGCGTAGACTTTG	3'	rev Fab Lc	60.19					
